# Supplementary material for: Synergism between IL7R and CXCR4 drives BCR-ABL induced transformation in Philadelphia chromosome-positive acute lymphoblastic leukemia
Source: Nat Commun. 2020 Jun 24;11:3194. doi: 10.1038/s41467-020-16927-w (PMC7314847; doi:10.1038/s41467-020-16927-w)
Supplement: Supplementary file 1 — Supplementary Information [file 41467_2020_16927_MOESM1_ESM.pdf]

Supplementary Information

Synergism between IL7R and CXCR4 drives BCR-ABL induced transformation in Philadelphia chromosome-positive acute lymphoblastic leukemia

Abdelrasoul et al.

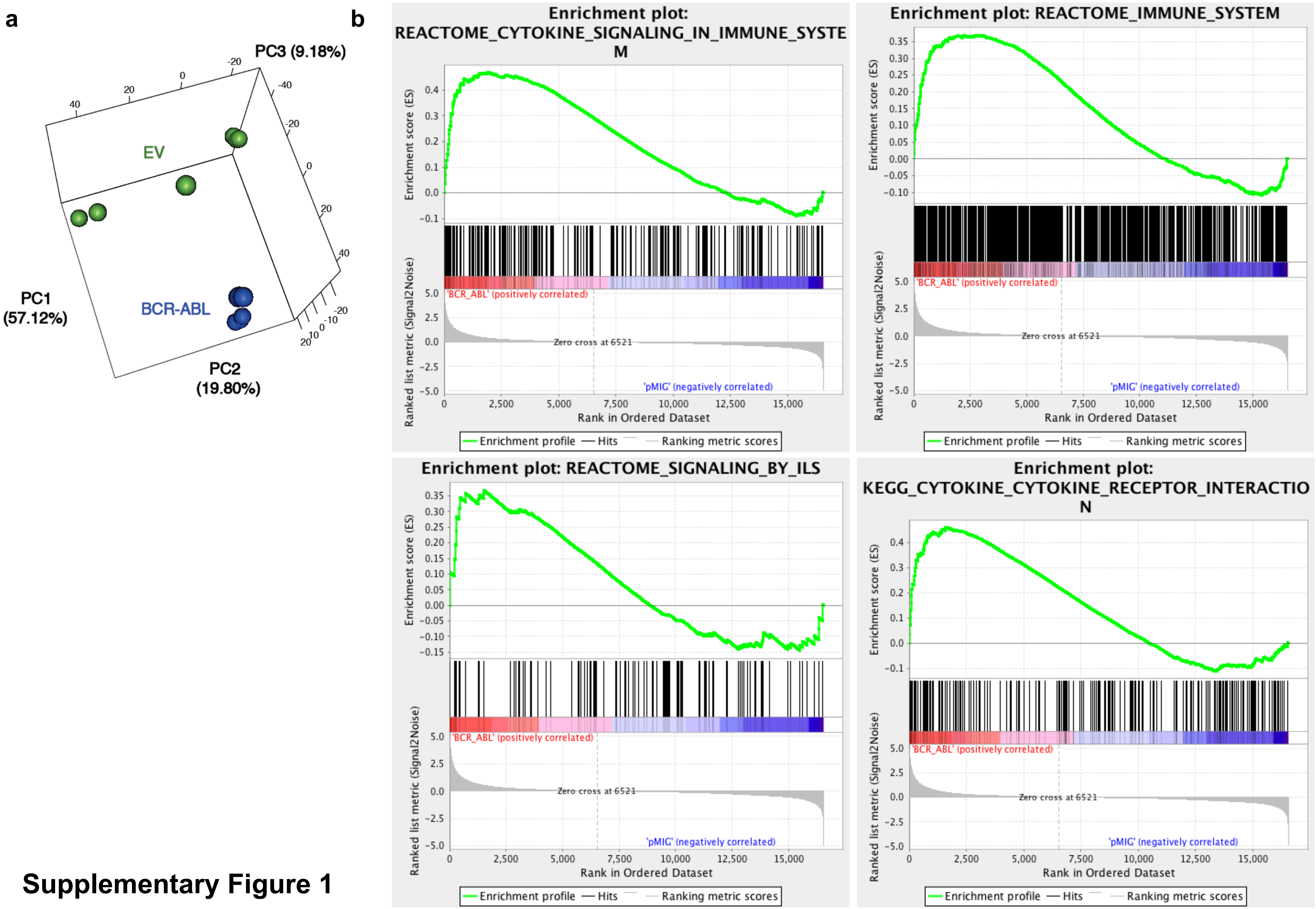

Supplementary Figure 1

Supplementary Figure 1: Upregulation of CXCR4 signaling pathway in BCR-ABL1-transformed pre-B cells.

(a) Gene expression variances between BCR-ABL1 transformed cell lines or control cell lines expressing empty vector (EV) are displayed as three-dimensional principal component analysis (PCA) of RNA-seq data. Each sample is represented as a dot and localized on basis of its gene expression pattern. PCA plot was used as an additional quality control for the RNA-seq data. Of note, EV and BCR-ABL samples show clear segregation on the first PC that explained 57.12% of the variation in the dataset. (b) Statistically significantly upregulated genesets related to IL7R signaling with False Discovery Rate (FDR) < 0.25; other than JAK-STAT pathway shown in Figure 1b. See Supplementary Table 2 for statistical details.

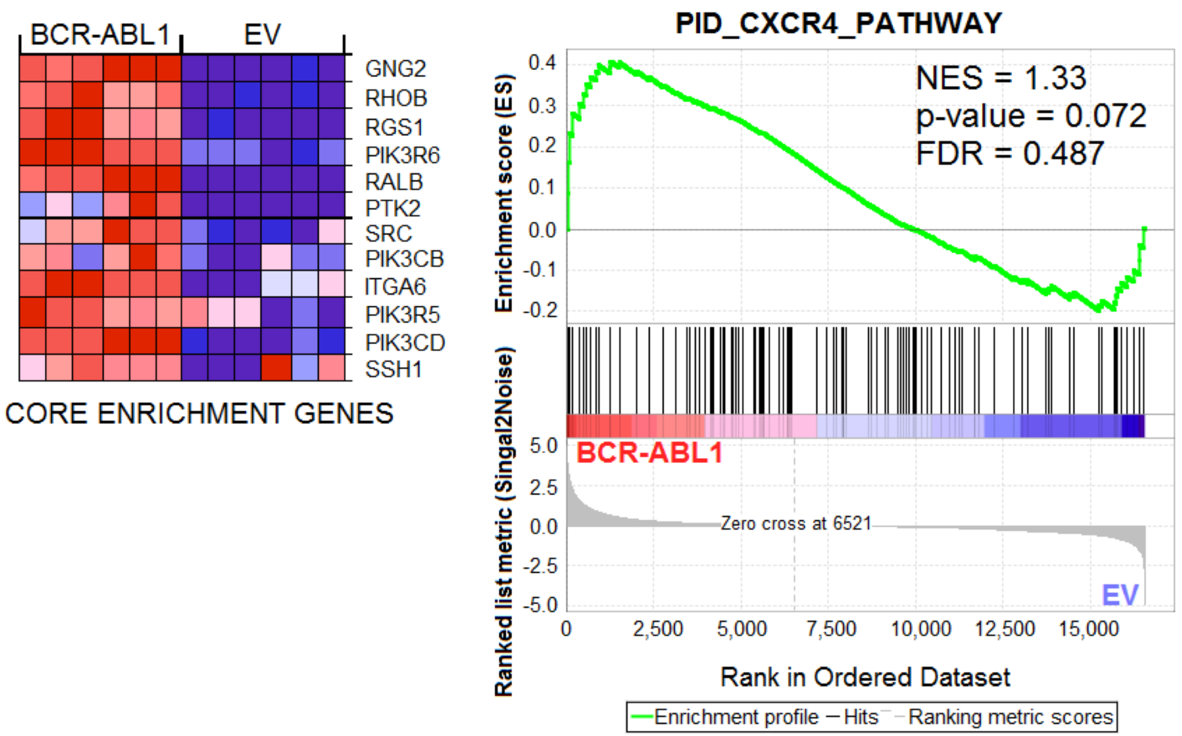

Supplementary Figure 2

Supplementary Figure 2: Upregulation of CXCR4 signaling pathway in BCR-ABL1-transformed pre-B cells. Gene Set Enrichment Analysis (GSEA) showing upregulation of gene set belonging to the CXCR4 signaling pathway in BCR-ABL1 group. Heatmap representation (left) of the top 12 deregulated genes (core enrichment genes) in BCR-ABL1 versus EV-transduced samples (Blue: down-regulated; Red: up-regulated; NES= Normalized Enrichment Score; FDR= False Discovery Rate). A two-sided signal-to-noise metric was used to rank the genes. For a calculated GSEA nominal p-values of 0, we present them as $p < 0.001$ (otherwise, exact p-values are shown). Multiple hypothesis testing correction is represented by the estimated FDR.

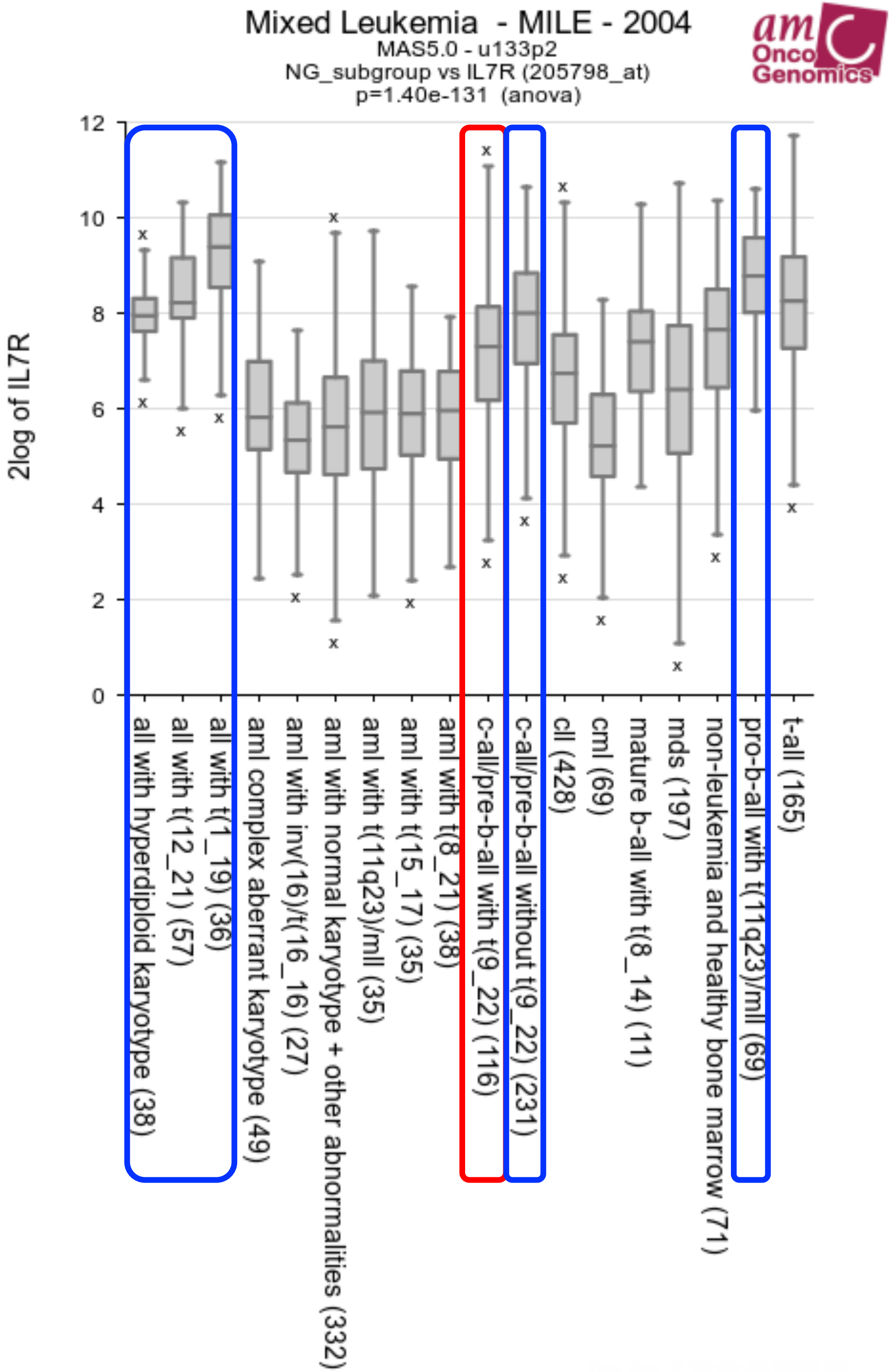

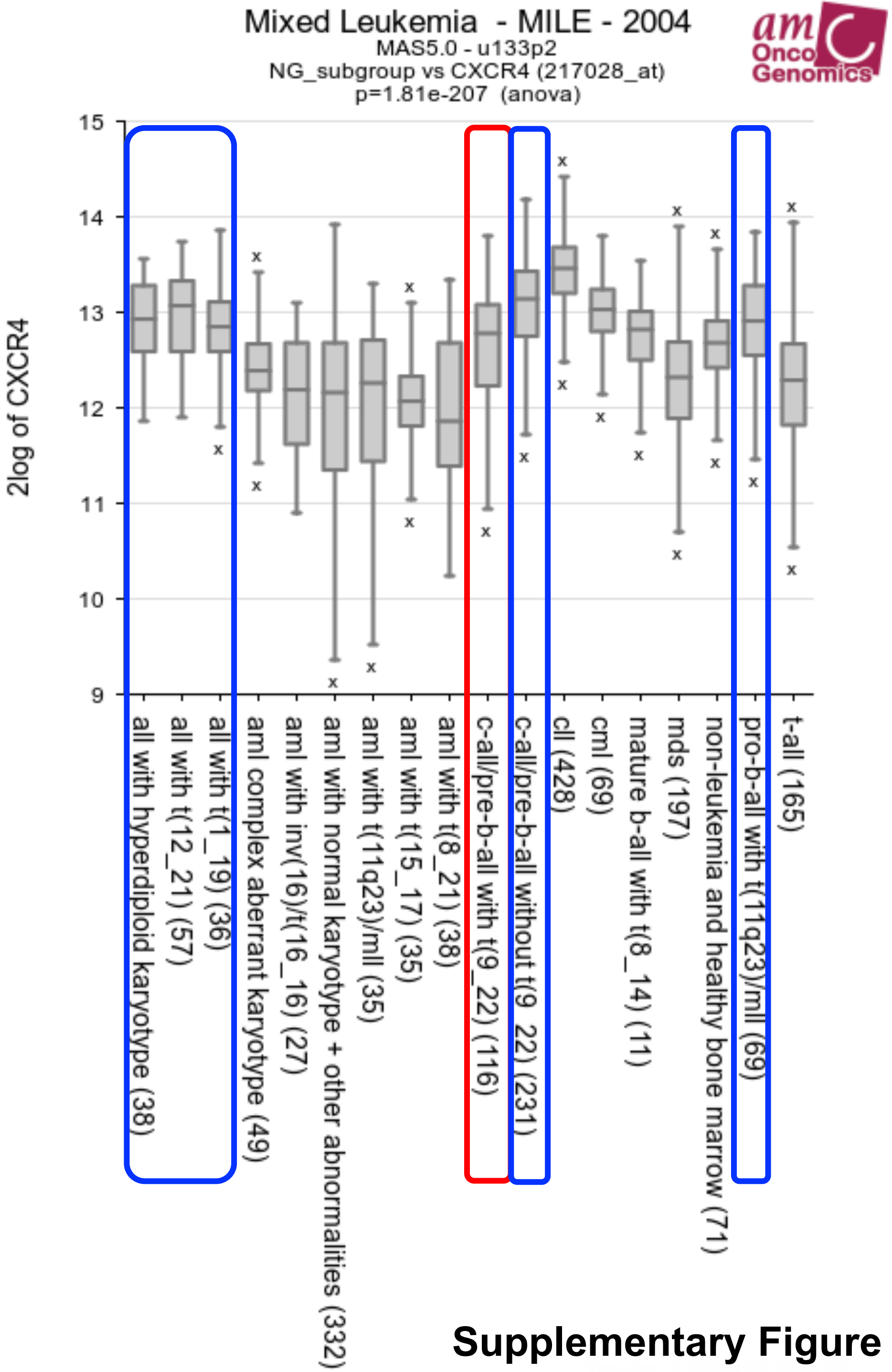

Supplementary Figure 3a

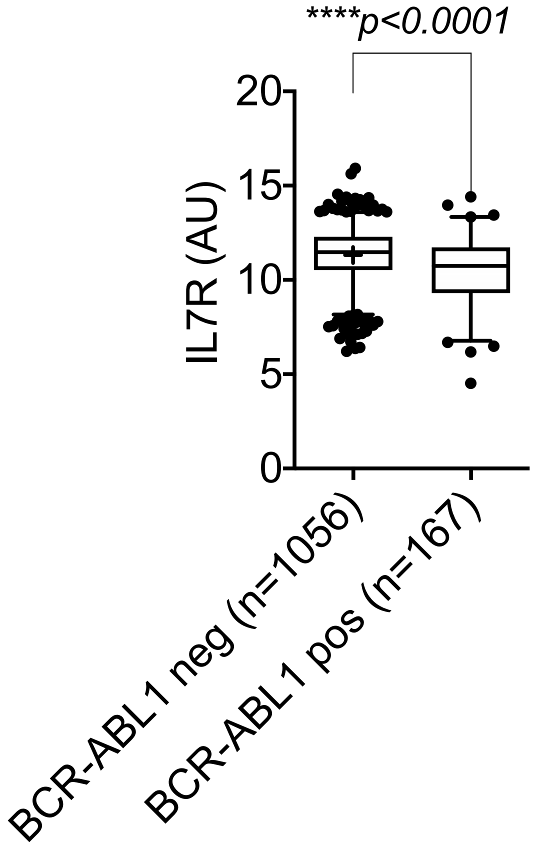

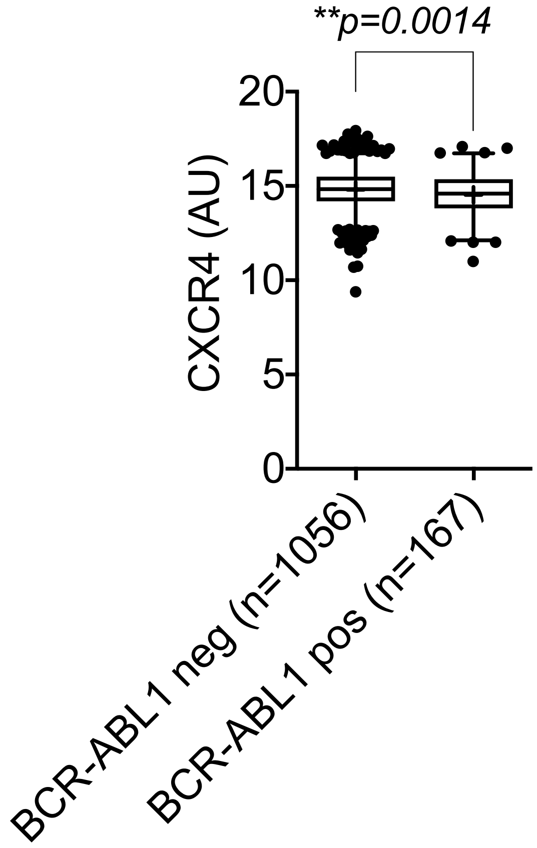

Supplementary Figure 3b

Supplementary Figure 3: Reduced IL7R and CXCR4 expression in BCR-ABL+ ALL as compared to other BCP-ALL subgroups.

(a) The expression levels of IL7R and CXCR4 in BCR-ABL+ ALL (t9; 22) (red) in comparison to other BCP-ALL entities (blue). The data are obtained from a mixed leukemia gene expression study[1]; R2 database. Box and whisker plots (25-75 percentile) are generated in R2 platform (https://hgserver1.amc.nl/cgi-bin/r2/main.cgi) (b) The expression levels of IL7R and CXCR4 in BCR-ABL positive ALL in comparison to other BCR-ABL negative BCP-ALL entities obtained from RNA-sequencing data of 1,223 patients.[2] Exact *p value for IL7R* ****$p = 0.000000000002$. (a-b) Values are depicted as box-whisker plots, the center line representing the median, box limits representing the 25th-75th percentile, whiskers depicting the 1.5x interquartile range and points representing outliers; unpaired, two-sided t-test. Additional information about the box plots are provided in Supplementary Table 4.

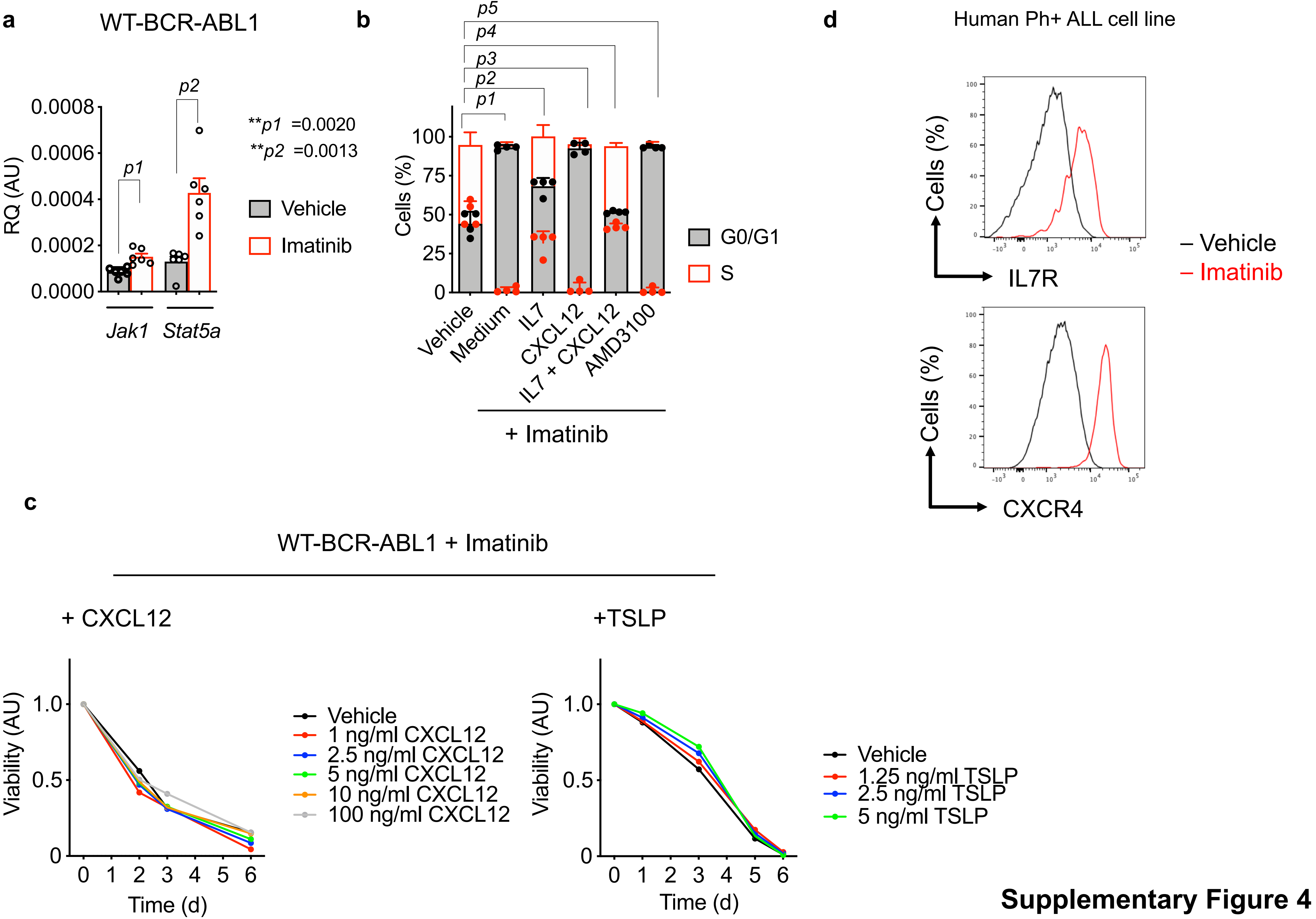

Supplementary Figure 4

Supplementary Figure 4: IL7 counteracts imatinib-induced cell death of BCR-ABL1⁺ cells.

(a) WT pre-B cells were transformed with BCR-ABL1 and were then treated with 5 μM imatinib for 15 hours. The expression levels of Jak1, and Stat5a were determined using qRT-PCR. N=6 per group, and error bars represent mean ± SD. Unpaired t-test, two-sided. (b) WT pre-B cells were transformed with BCR-ABL1 and were then treated with 1 μM imatinib for 15 hours in the presence of IL7, or CXCL12, or both IL7 and CXCL12, or with AMD3100 as indicated, then cell cycle analysis was performed. Quantification of percentages of cells in S-phase or G0/G1 is shown. AU: arbitrary unit. (n=4 per group), error bars represent mean ± SD. Unpaired t-test, two-sided. G0/G1 (**p1=0.0286*, **p2=0.0286*, **p3=0.0286*, **p4=0.0286*, n.s. *p5=0.8750*); S phase (**p1=0.0286*, **p2=0.0286*, **p3=0.0286*, **p4=0.0286*, n.s. *p5=0.8750*). (c) BCR-ABL1-transformed WT pre-B cells were treated with 1 μM imatinib and with CXCL12 (left) or TSLP (right) at different concentrations as indicated for 6 days. The ratio of living cells was determined by flow cytometry. AU: arbitrary unit. (n=2 per group). (d) Human Ph+ ALL SUP-B 15 cell line was treated with 5 μM imatinib and surface expression of IL7R (top) and CXCR4 (bottom) was measured by flow cytometry at day 7. The results are representative of 3 independent experiments.

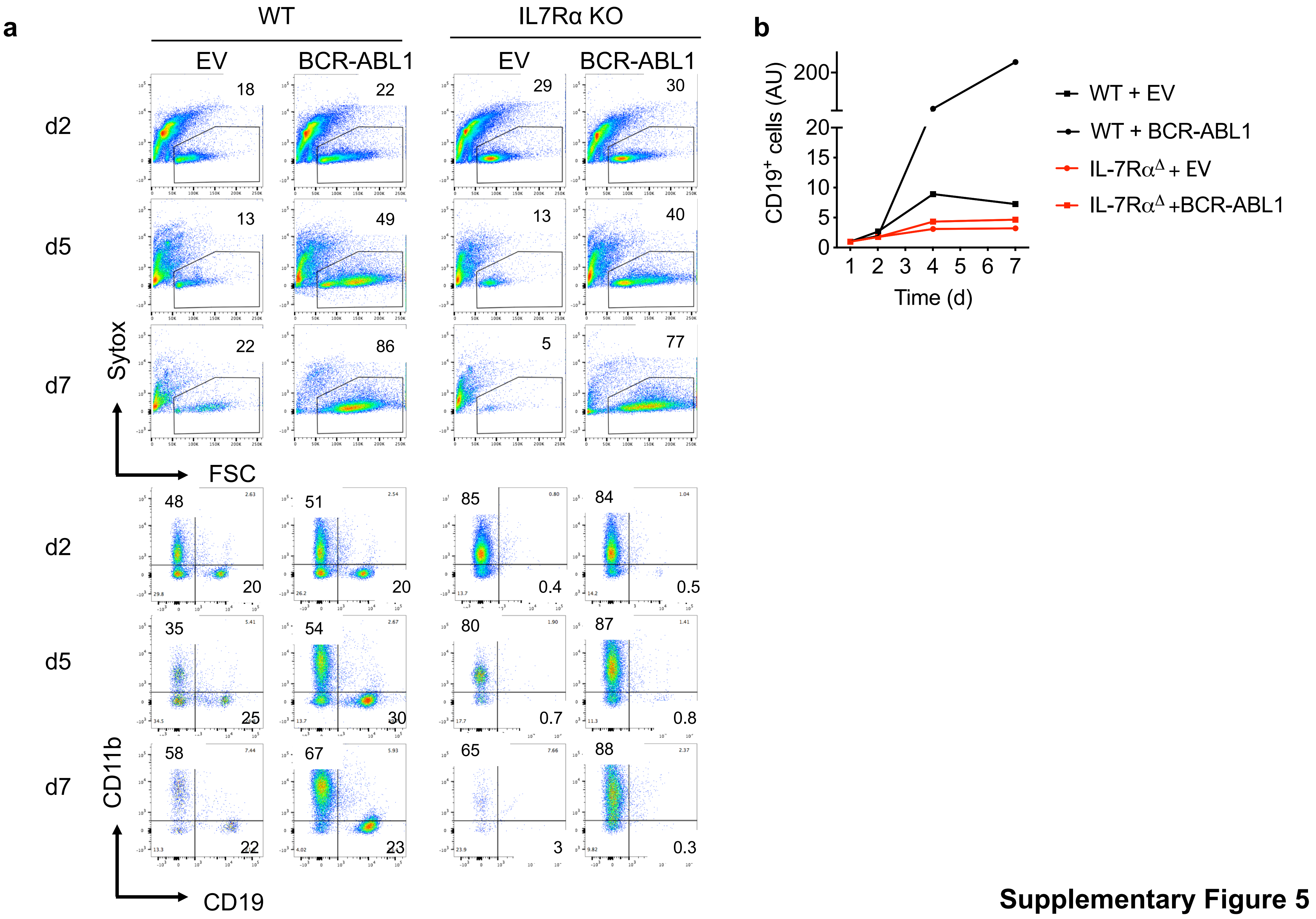

Supplementary Figure 5

Supplementary Figure 5: IL7R is indispensable for BCR-ABL1-derived transformation.

(a) Bone marrow-derived pre-B cells from either WT or IL7Rα knock out (KO) mice were transduced with either an empty vector (EV) or with BCR-ABL1. Viability of the cells after IL7 withdrawal was determined by flow cytometry (top) using Syotx as excluding dead cell stain. The percentages of lymphoid ($CD19^{+}GFP^{+}$) or myeloid ($CD11b^{+}GFP^{+}$) cells were analyzed (bottom). (b) The enrichment of $CD19^{+}$ cells in relative to control after IL7 withdrawal (n=2 per group). AU: arbitrary unit.

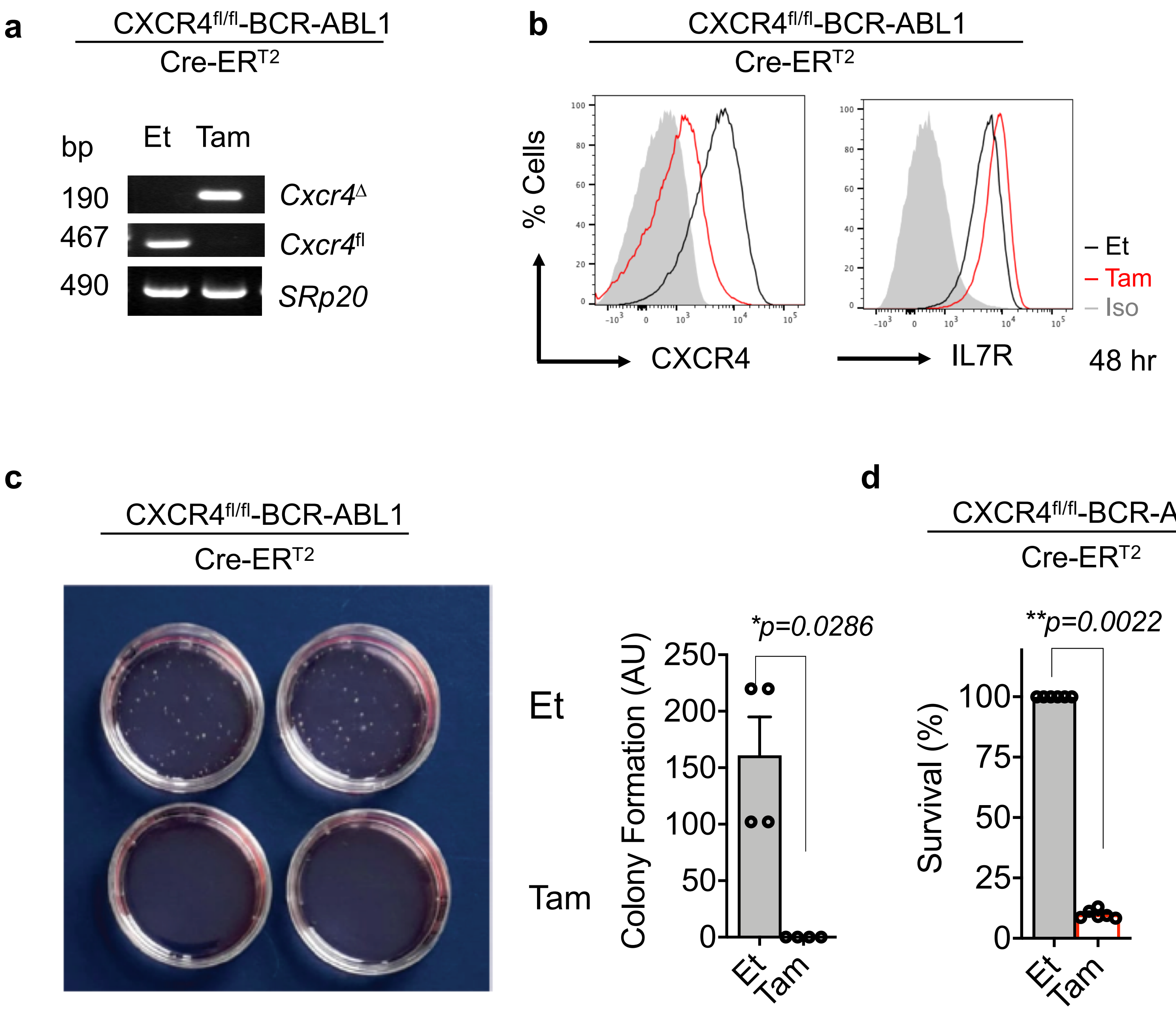

Supplementary Figure 6

Supplementary Figure 6: CXCR4 expression is required for the survival of BCR-ABL1 transformed cells.

(a) $CXCR4^{fl/fl}$ pre-B cells were transformed with BCR-ABL1 and were then transduced with $Cre\text{-}ER^{T2}$. Cells were treated with either tamoxifen (Tam) to induce Cre expression, or with ethanol (Et). PCR analysis for CXCR4 deletion ($CXCR4^{\Delta}$), SRp20 was used as a loading control. The results are representative of 3 independent experiments; bp: base pair. (b) The surface expression of IL7R or CXCR4 after 24 hours of Tam induction. (c) Colony formation assay for $CXCR4^{fl/fl}$ cells which were transduced with BCR-ABL1 and $Cre\text{-}ER^{T2}$. Cells were treated with either Et or Tam and incubated to allow colony formation for 3 weeks. N=4 independent samples per group, and error bars represent mean ± SD. Unpaired t-test, two-sided. (d) The percentage of living cells were determined by flow cytometry using Sytox as an excluding dead cell stain, after 72 hours of tamoxifen induction. N=6 independent samples per group, and error bars represent mean ± SD. Unpaired t-test, two-sided.

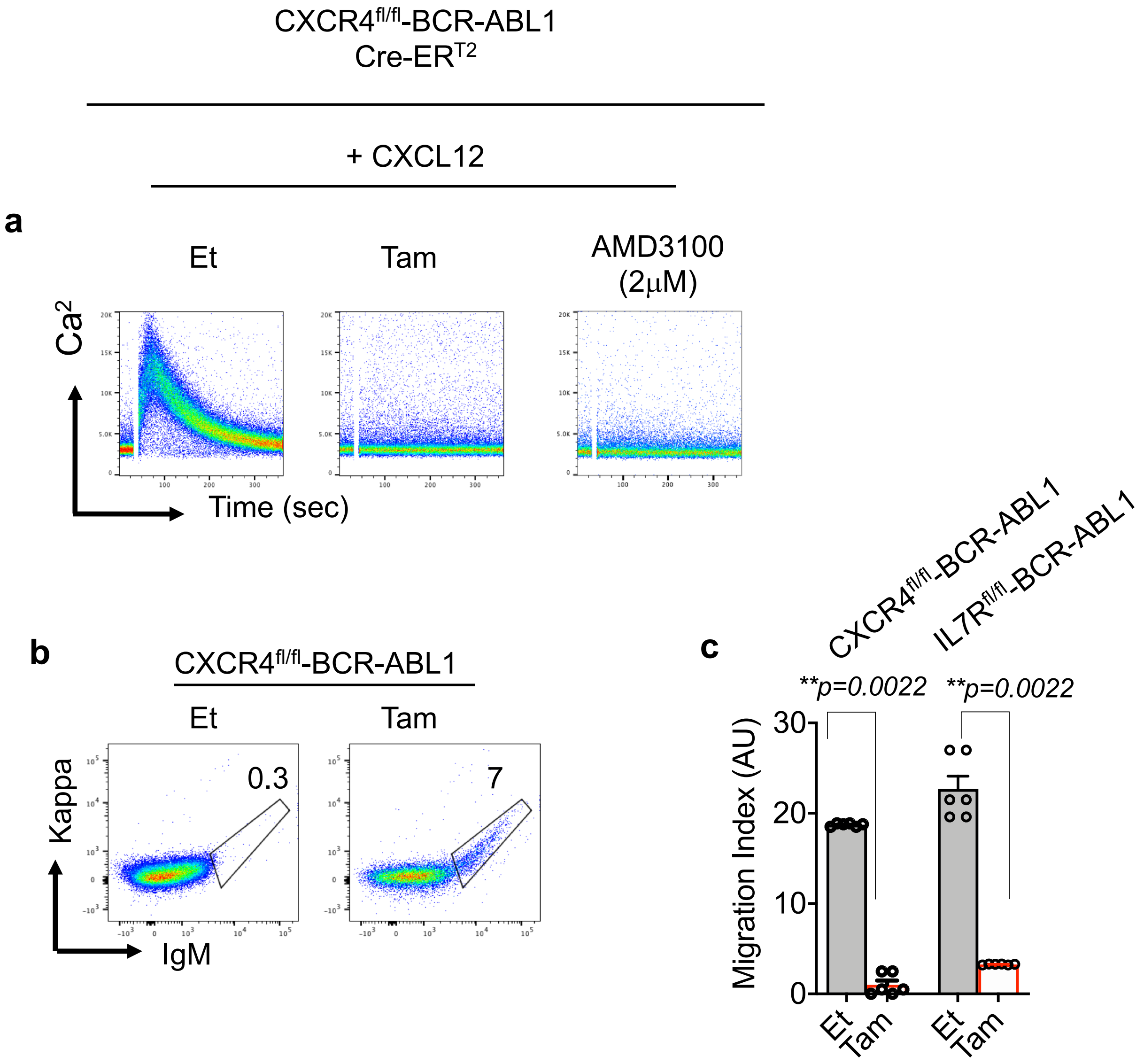

Supplementary Figure 7

Supplementary Figure 7: CXCR4 role in calcium mobilization and migration of BCR-ABL1 transformed cells.

(a) $CXCR4^{fl/fl}$-BCR-ABL1 cells containing Cre-ER^{T2} were induced with either ethanol (Et) or tamoxifen (Tam) for 48 hours, then CXCL12-induced calcium flux was measured. CXCR4 inhibition using AMD310 was used as a control. The results are representative of 3 independent experiments. (b) Enrichment of differentiated cells (µ+kappa+) in CXCR4 deficient cells (n=2). (c) CXCR4 or IL7Rα deletion was induced by tamoxifen for 24 hours and cells were then subjected to migration gradient toward CXCL12 (100ng/ml) for 16 hours. The cells in the lower chamber were counted by trypan blue. N=6 per group, error bars represent mean ± SD. Unpaired t-test, two-sided.

Supplementary Figure 8

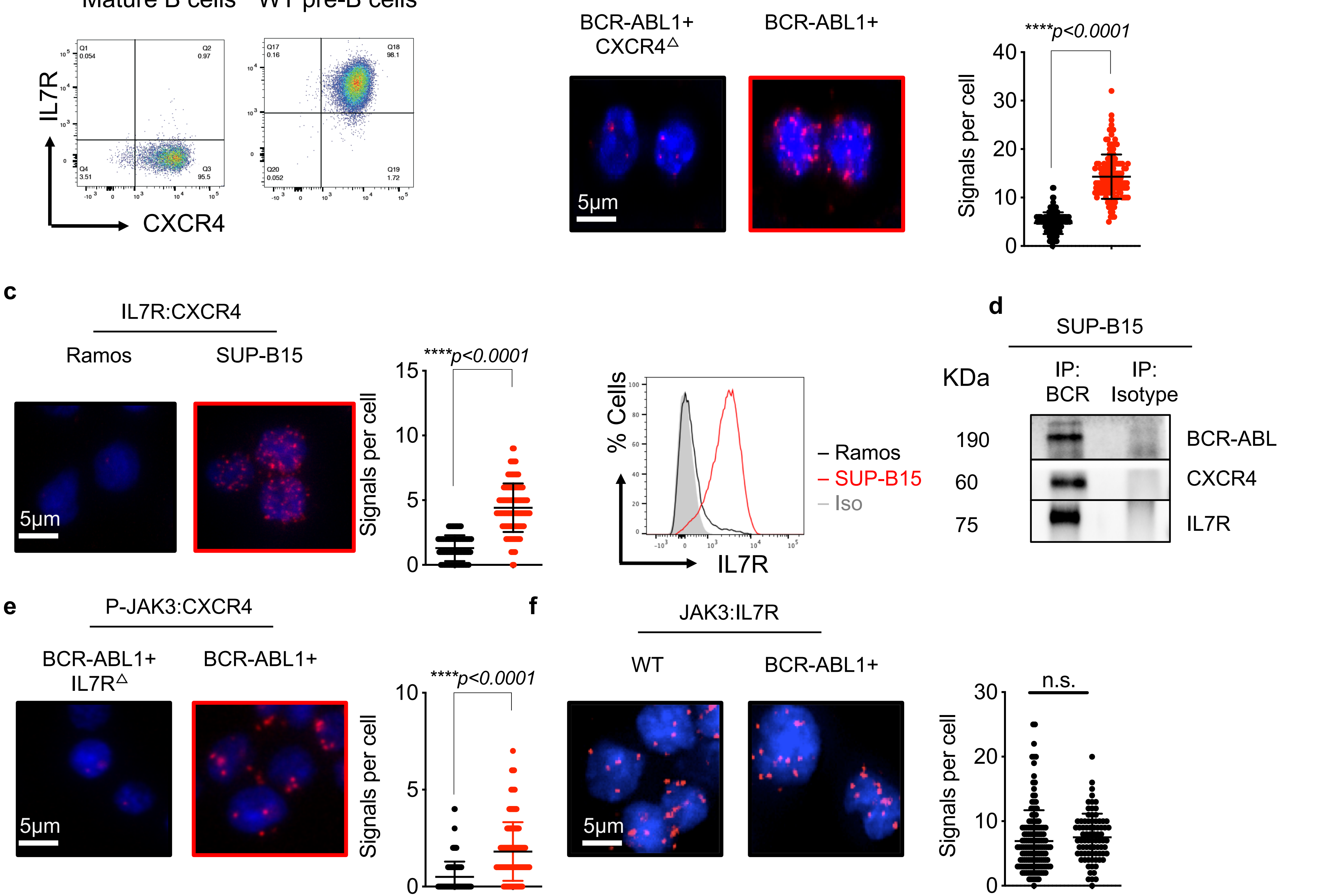

Supplementary Figure 8: CXCR4 and IL7R exist in close proximity in both mouse and human BCR-ABL1^{+}ALL cells.

(a) Flow cytometry staining of bone marrow-derived pre-B cells or mature splenic B cells which were used for PLA experiments in Figure 4b. The results are representative of 3 independent experiments. (b) PLA showing the loss of close proximity between IL7R and CXCR4 in BCR-ABL1 transformed cells upon inducible deletion of CXCR4 using Cre-ERT2 system. Quantification of red dots (right). (c) Fab-PLA analysis of IL7R-CXCR4 proximity in in human BCR-ABL+ cell line (SUP-B15) in comparison to Ramos cells. Quantification of red dots (middle). IL7R surface expression as measured by flow cytometry (left). (d) SUP-B15 Ph+ ALL cells were lysed and an Immunoprecipitation (IP) was performed using an antibody against the BCR. IP with an isotype antibody was used as a negative control. The results are representative of 2 independent experiments; KDa: Kilo Dalton (e) PLA showing that close association between p-Jak3 and CXCR4 is reduced in BCR-ABL1 transformed cells upon inducible deletion of IL7R using Cre-ERT2 system. (f) PLA showing that close association between Jak3 and IL7R is not significantly different between WT pre-B cells and BCR-ABL1 transformed cells. (b-c, e-f) Quantification shows number of signals per cell, error bars represent mean ± SD. Unpaired t-test, two-sided. The results are representative of 3 independent experiments.

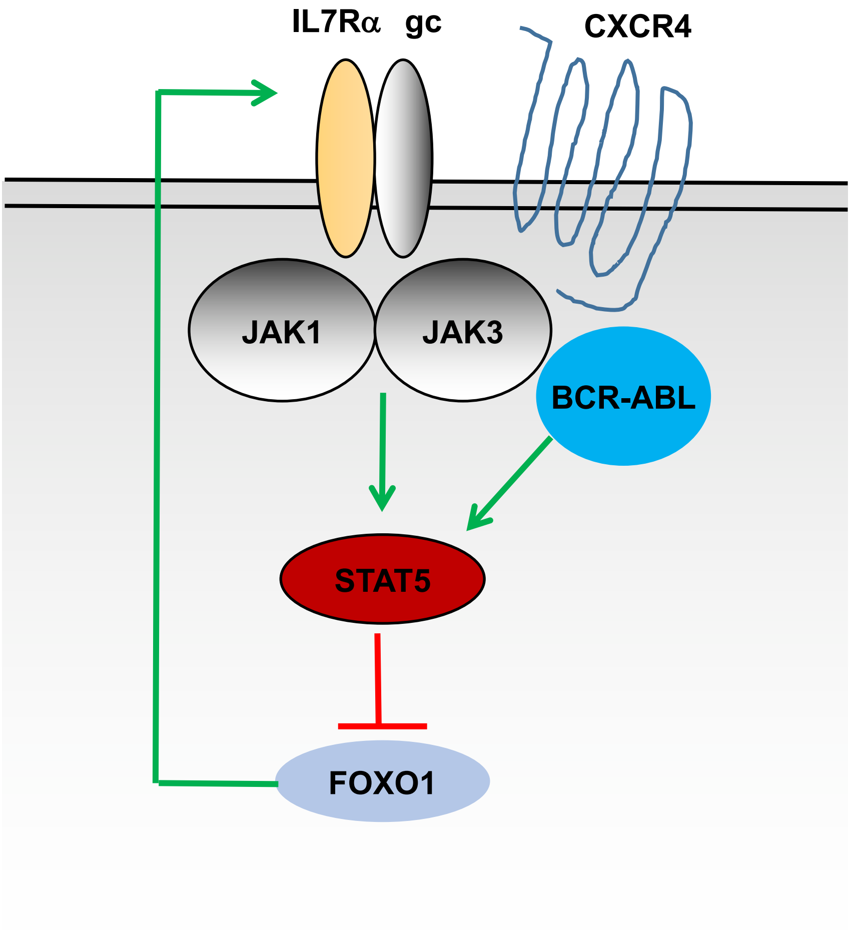

Supplementary Figure 9

Supplementary Figure 9: Simplified graph for the proposed regulatory model.

IL7R and CXCR4 synergize to facilitate BCR-ABL1-mediated pre-B cell transformation. In this platform, CXCR4 recruits BCR-ABL1 into close proximity to the IL7R downstream signaling pathway (JAK/STAT) which results in STAT5 activation. STAT5 activation inhibits FOXO1 transcription factor, which in turn regulates IL7R expression.

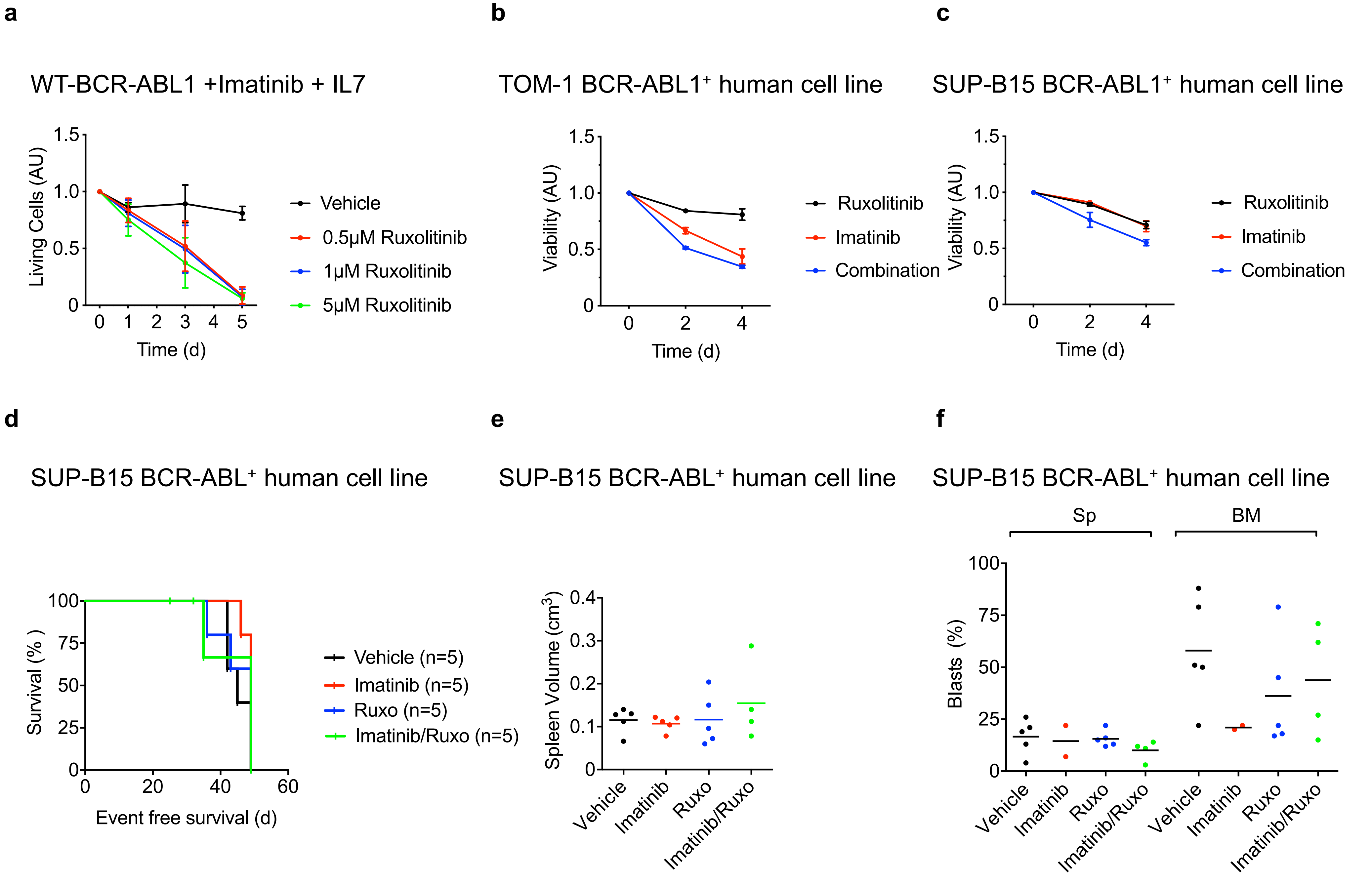

Supplementary Figure 10

Supplementary Figure 10: Effects of ruxolitinib treatment in BCR-ABL+ ALL.

(a) WT pre-B cells transformed with BCR-ABL1 were treated with 1μM imatinib and 2.5ng/ml IL7 in the presence of different concentrations of ruxolitinib and the fold change of living cells relative to control was determined by flow cytometry. N=3 independent samples per group, and error bars represent mean ± SD. One-way ANAOVA. Dunnett's multiple comparisons test was performed to day 5, compared to control group (Vehicle). Adjusted *p* values: Veichle vs. 0.5μM Ruxolitinib ****$p<0.0001$, Veichle vs. 1μM Ruxolitinib ****$p<0.0001$, Veichle vs. 5μM Ruxolitinib****$p<0.0001$. Human BCR-ABL1+ cell lines TOM-1 (b) and SUP-B15 (c) were treated with vehicle only, imatinib only (2μM), ruxolitinib only (10μM) or combination of ruxolitinib and imatinib and the percentage of living cells were determined using Sytox viability dye (n=3 per group) relative to control. (b-c) N=3 independent samples per group, and error bars represent mean ± SD. One-way ANAOVA. Dunnett's multiple comparisons test was performed to day 5, compared to control group (Vehicle). Adjusted p values (b): Vehicle vs. Imatinib **p *=0,0014*, Vehicle vs. Ruxo****$p<0.0001$, Vehicle vs. Imatinib/Ruxo****$p<0.0001$. Adjusted p values (c): Vehicle vs. Imatinib ***$p<0.0001$, Vehicle vs. Ruxo****$p<0.0001$, Vehicle vs. Imatinib/Ruxo****$p<0.0001$. (d) SUP-B15 ALL cells were injected into NSG mice and the mice were subjected to treatment with vehicle only, imatinib only, ruxolitinib only or combination of ruxolitinib and imatinib (n=5 per group) as described in Methods. Survival prolongation in xenografted mice subjected to the indicated treatment. Statistics for survival were performed according to the Mantel-Cox log-rank method and showed no significant statistical difference among the groups (e) Spleen sizes from the corresponding groups. (f) Leukemic engraftment was measured by flow cytometry in BM and spleen. Unpaired t-test, two-sided, no significant statistical difference among the groups when compared to control group (vehicle).

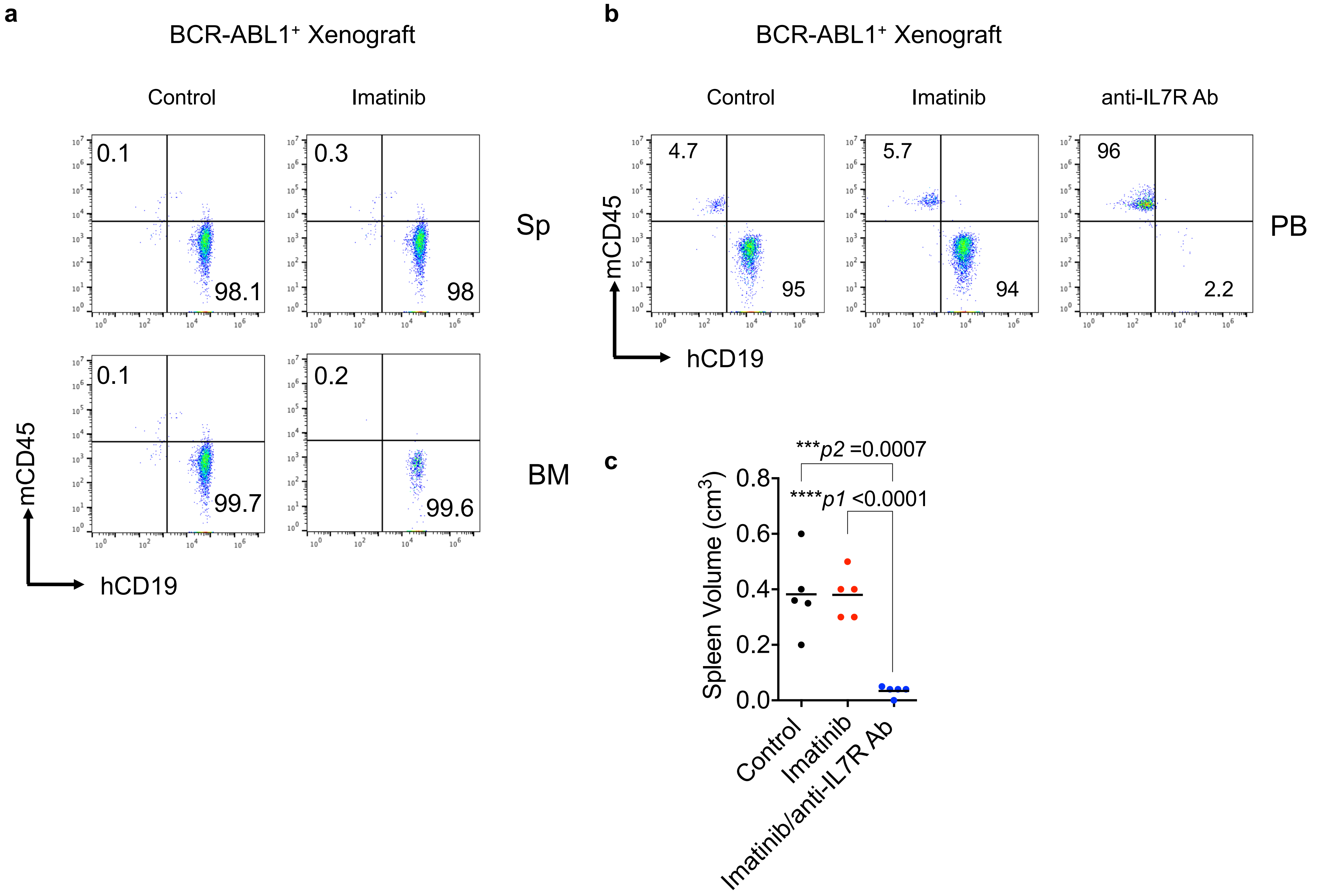

Supplementary Figure 11

Supplementary Figure 11: *in vivo* engraftment of imatinib-resistant BCR-ABL$^+$ ALL

Xenograft mice of an imatinib-resistant BCR-ABL patient material were generated and treated with either vehicle or imatinib (n=5 per group) as described in Figure7a. (a) A representative flow cytometry staining of leukemic blasts derived from bone marrow (BM) or spleen (Sp) or (b) peripheral blood at day 58. The percentage of leukemic cells (positive for human CD19) are indicated in the lower right quadrant of each plot. (c) Spleen size of xenografted mice treated with imatinib or with imatinib and IL7R antibody as described in Figure 7f. Unpaired t-test, two-sided.

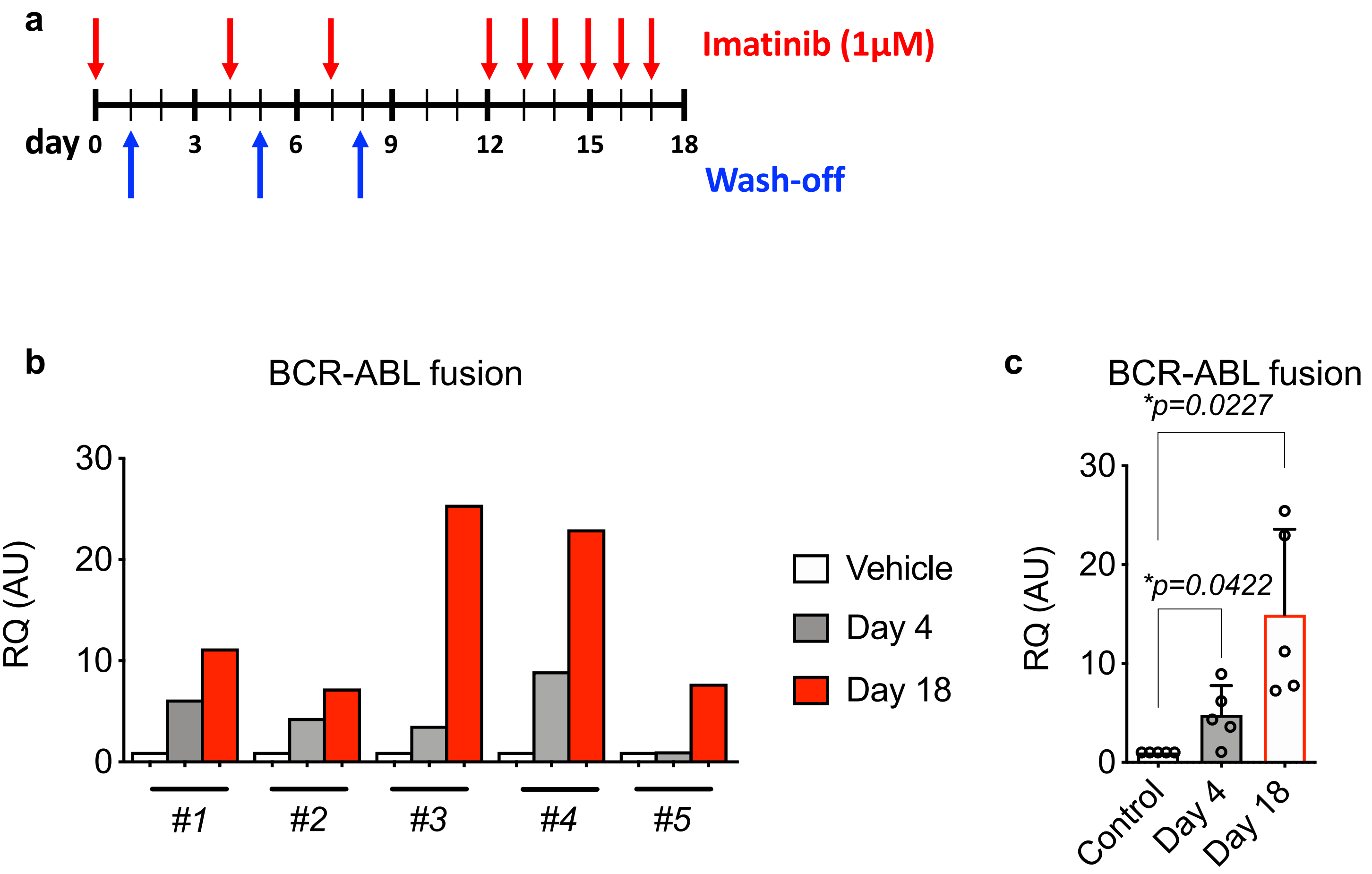

Supplementary Figure 12

Supplementary Figure 12: Upregulation of BCR-ABL can lead to imatinib-resistance in BCR-ABL$^+$ transformed pre-B cells.

Mouse WT pre-B cells transformed with BCR-ABL1 were treated with either vehicle or imatinib (1µM) for 18 days to induce imatinib-resistance. (a) A schematic diagram showing the time schedule for adding or washing-off imatinib. (b) Expression levels of BCR-ABL1 fusion in five different WT-BCR-ABL transformed pre-B cells as measured by quantitative RT-PCR. Expression levels are normalized to cells treated with vehicle (DMSO). (c) A summary graph for the upregulation of BCR-ABL of all cell lines. Paired t-test, two-sided p value.

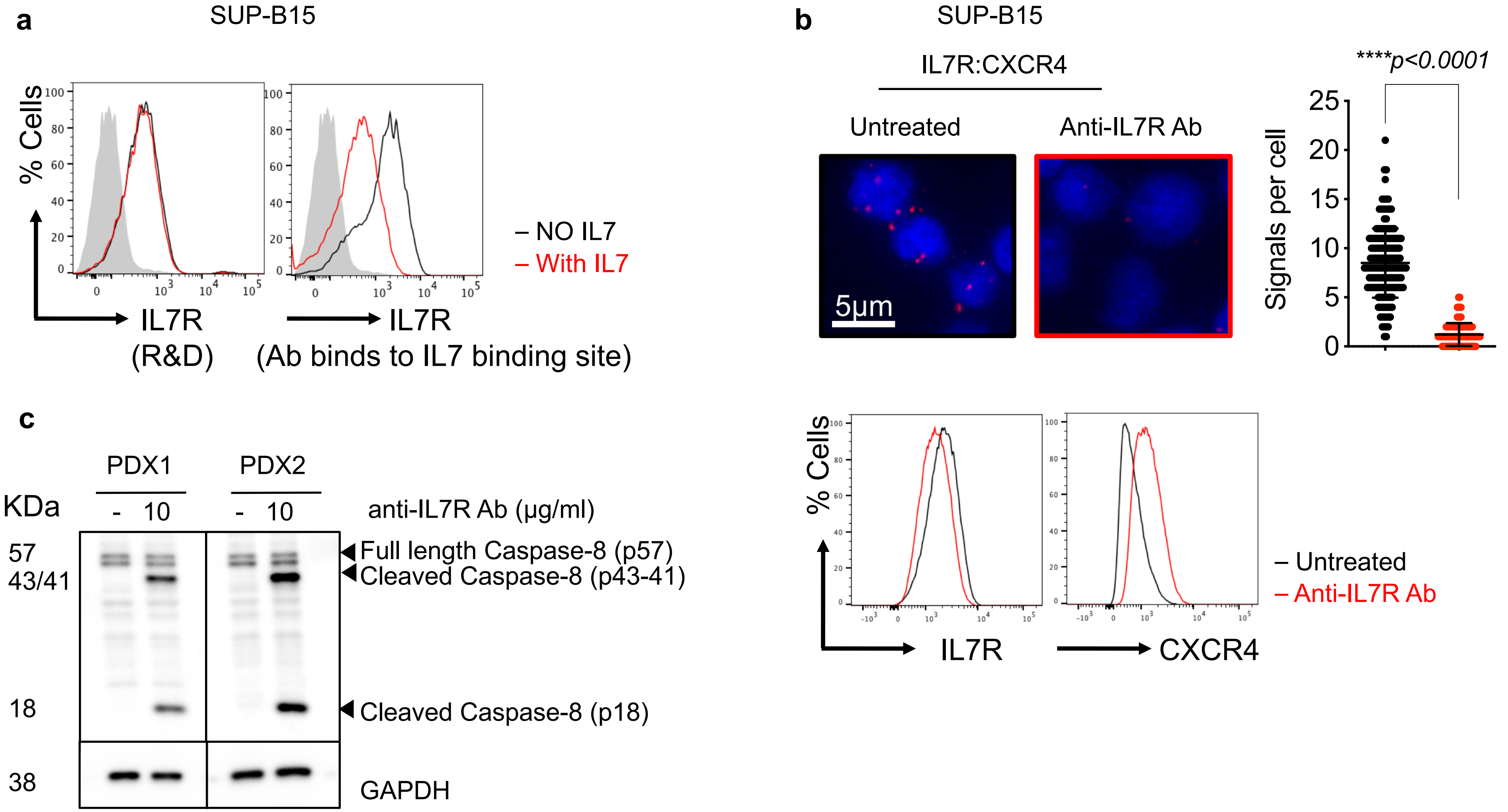

Supplementary Figure 13

Supplementary Figure 13: Anti-IL7R antibody induces apoptosis. (a) A representative extracellular flow cytometry staining showing that anti-IL7R antibody used for *in vivo* treatment (in Figure 7) does not bind to the ligand binding site of IL7R. Sup-B15 cells were incubated with human IL7 for 15 minutes, then were treated with two different anti-IL7R antibodies (left: from R&D; right: from BioLegend) for 15 minutes on ice. A secondary antibody was used when required. (b) Fab-PLA analysis of extracellular IL7R-CXCR4 proximity in SUP-B15 which were treated with 10µg/ml anti-IL7R antibody for 45 minutes at 37°C in comparison to untreated cells. Quantification of red dots shows number of signals per cell (top), error bars represent mean ± SD. Unpaired t-test, two-sided, **** $p<0.000000000000001$. IL7R and CXCR4 surface expression as measured by flow cytometry after the treatment (bottom). The results are representative of 3 independent experiments. (c) Anti-IL7R antibody treatment leads to apoptosis. Two different BCR-ABL1+ patient derived xenograft (PDX) ALL cells were treated 10µg of anti-IL7R antibody for 45 minutes. Cell lysates were subjected to western blotting and Caspase-8 levels were detected. Treatment with anti-IL7R antibody activates caspase-8 cleavage and leads to the release of the caspase-8 active fragment p18. The results are representative of 2 independent experiments. KDa: Kilo Dalton.

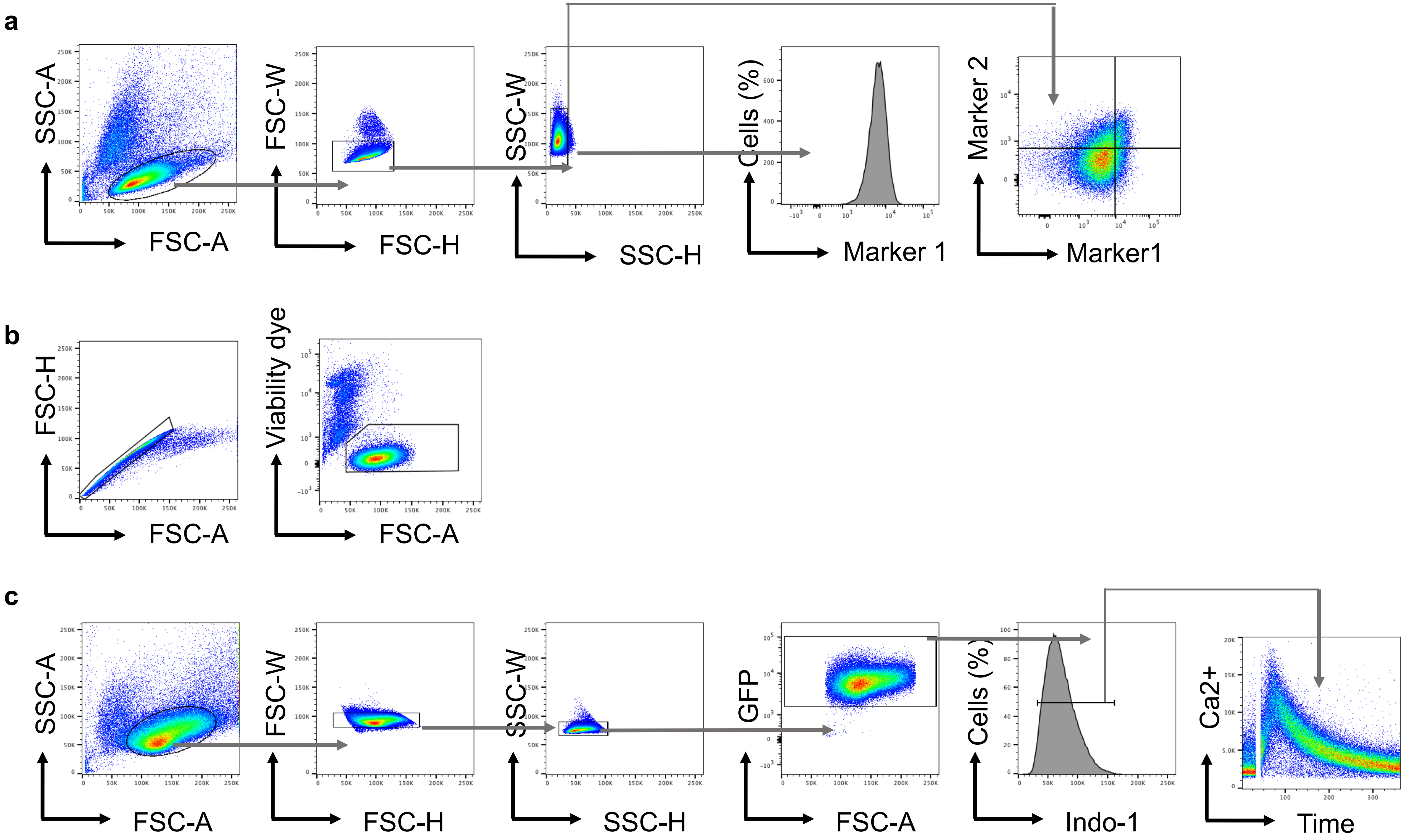

Supplementary Figure 14

Supplementary Figure 14: Gating strategies used for flow cytometry analysis. (a) Lymphocytes gate was analyzed depending on distinguished FSC vs. SSC properties. Singlets were then selected (FSC-W vs FSC-H then SSC-W vs SSC-H). The cells were then further analyzed according to their surface or intracellular protein stains. Similar gating strategy was applied to Figures: 2b, 3a, 3d, 5c, 5e, 6d, 7b, 7e, Supp 4b, 4d, Supp 6b, Supp 7b, Supp 8a, 8c, Supp 10f, Supp 11a-b,and Supp 13a-b.

(b) For viability experiments, singlets were first selected (FSC-A vs FSC-H). Viable cells were then determined according to FSC-A and viability dye properties. Similar gating strategy was applied to Figures: 2d-e, 3b, 3e, 6b-c, Supp 4c, Supp 5, Supp 6d,and Supp 10a-c.

(c) For calcium analysis, lymphocytes gate was analyzed depending on distinguished FSC vs. SSC properties. Singlets were then selected (FSC-W vs FSC-H then SSC-W vs SSC-H). The cells stained with indo-1 were then selected and then calcium kinetics were then shown relative to time. Similar gating strategy was applied to Figures: 4a, and Supp 7a.

Original Gel Blots for Fig 4e (left panel)

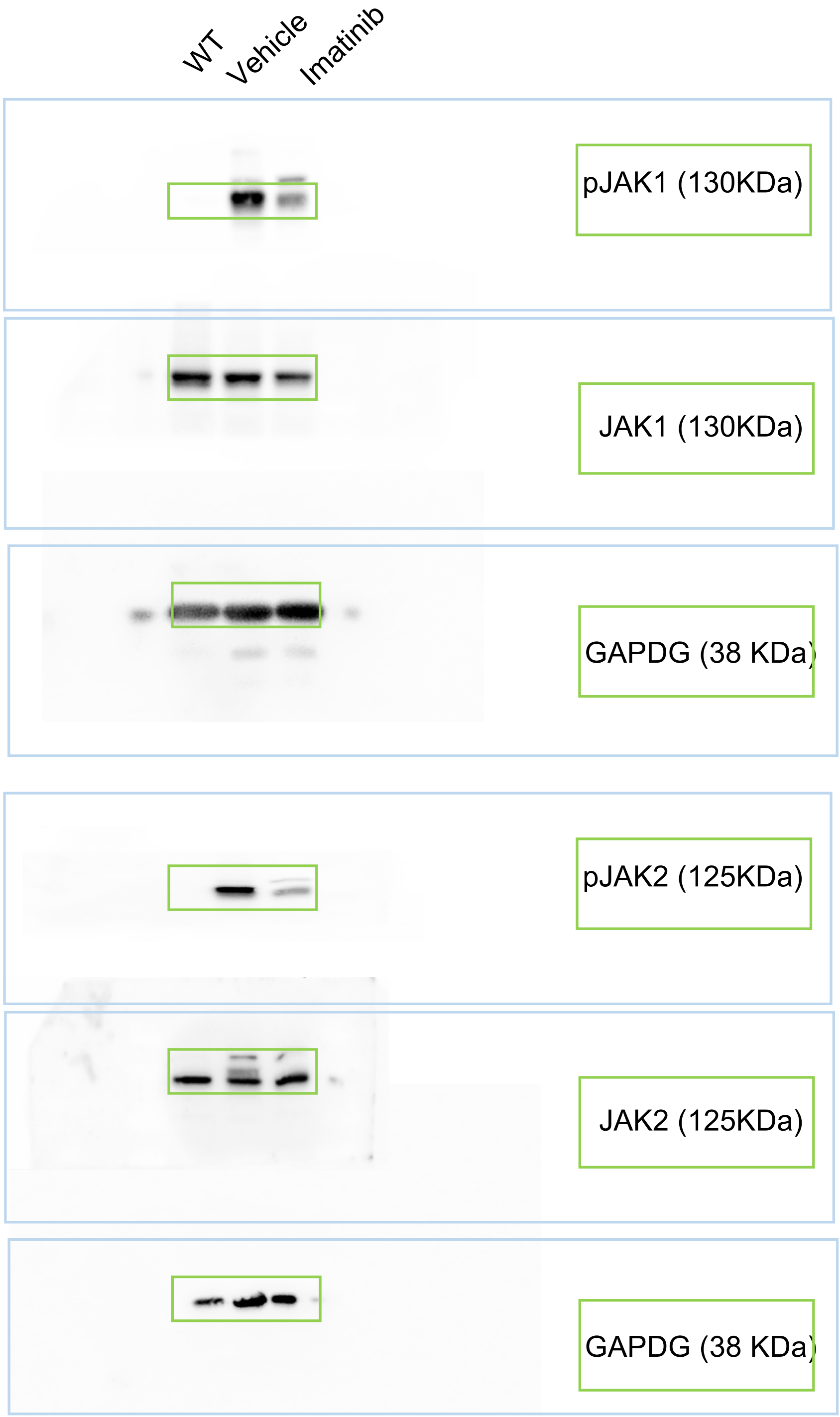

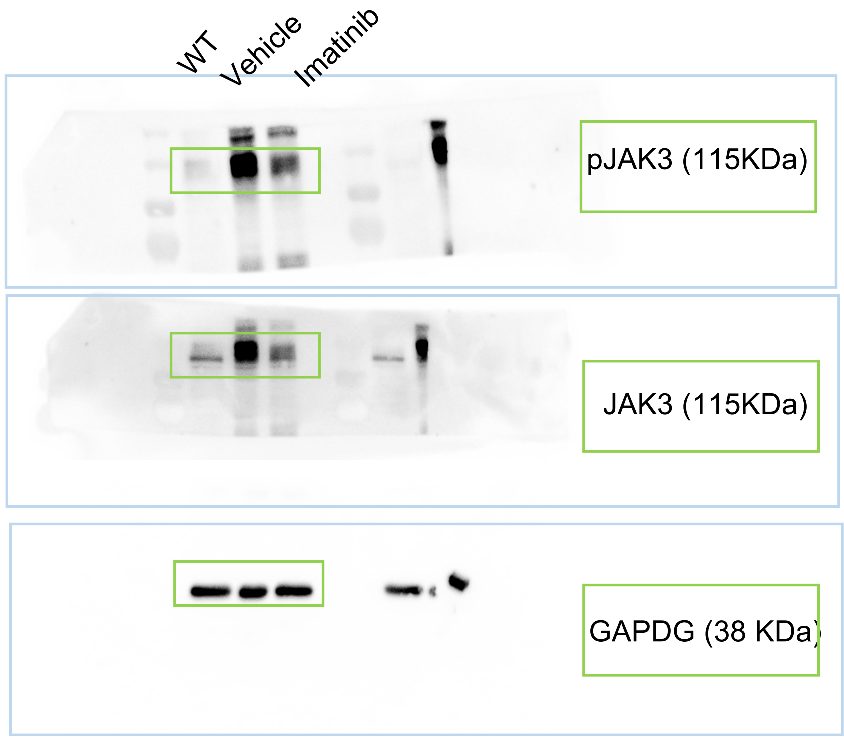

Supplementary Figure 15a

Original Gel Blots for Fig 4e (right panel)

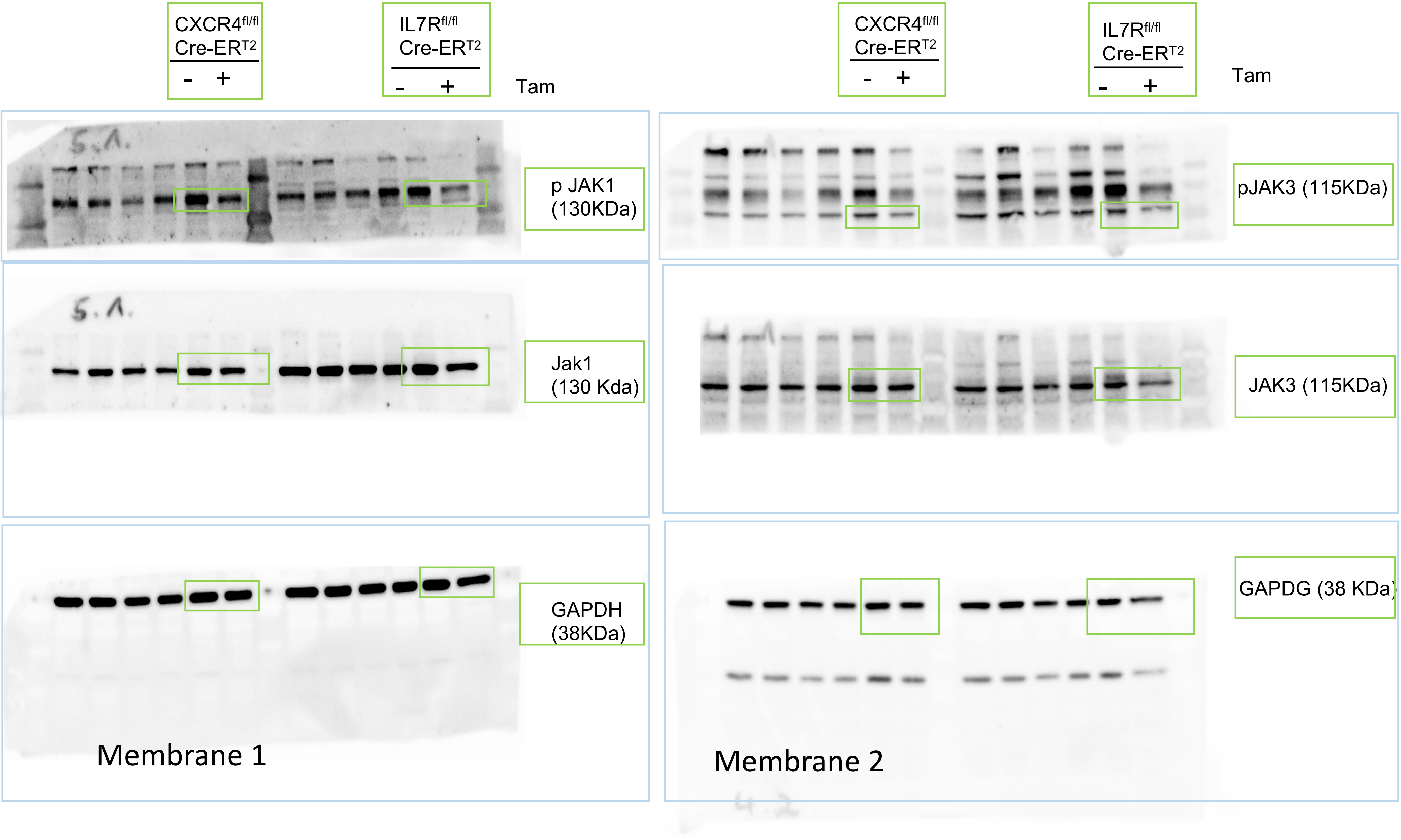

Supplementary Figure 15a continued

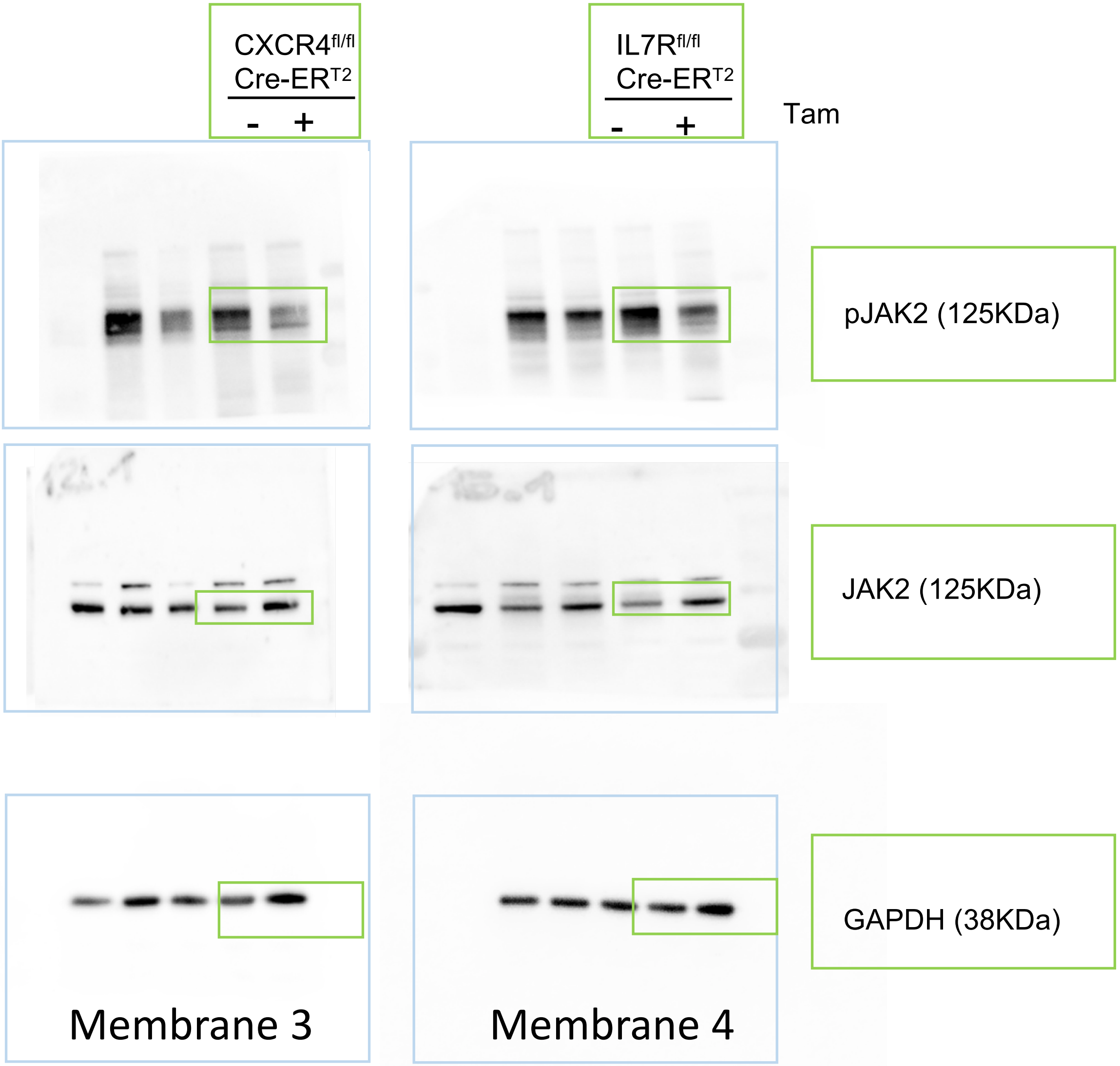

Supplementary Figure 15a continued

Original Gel Blots
for Fig 5a

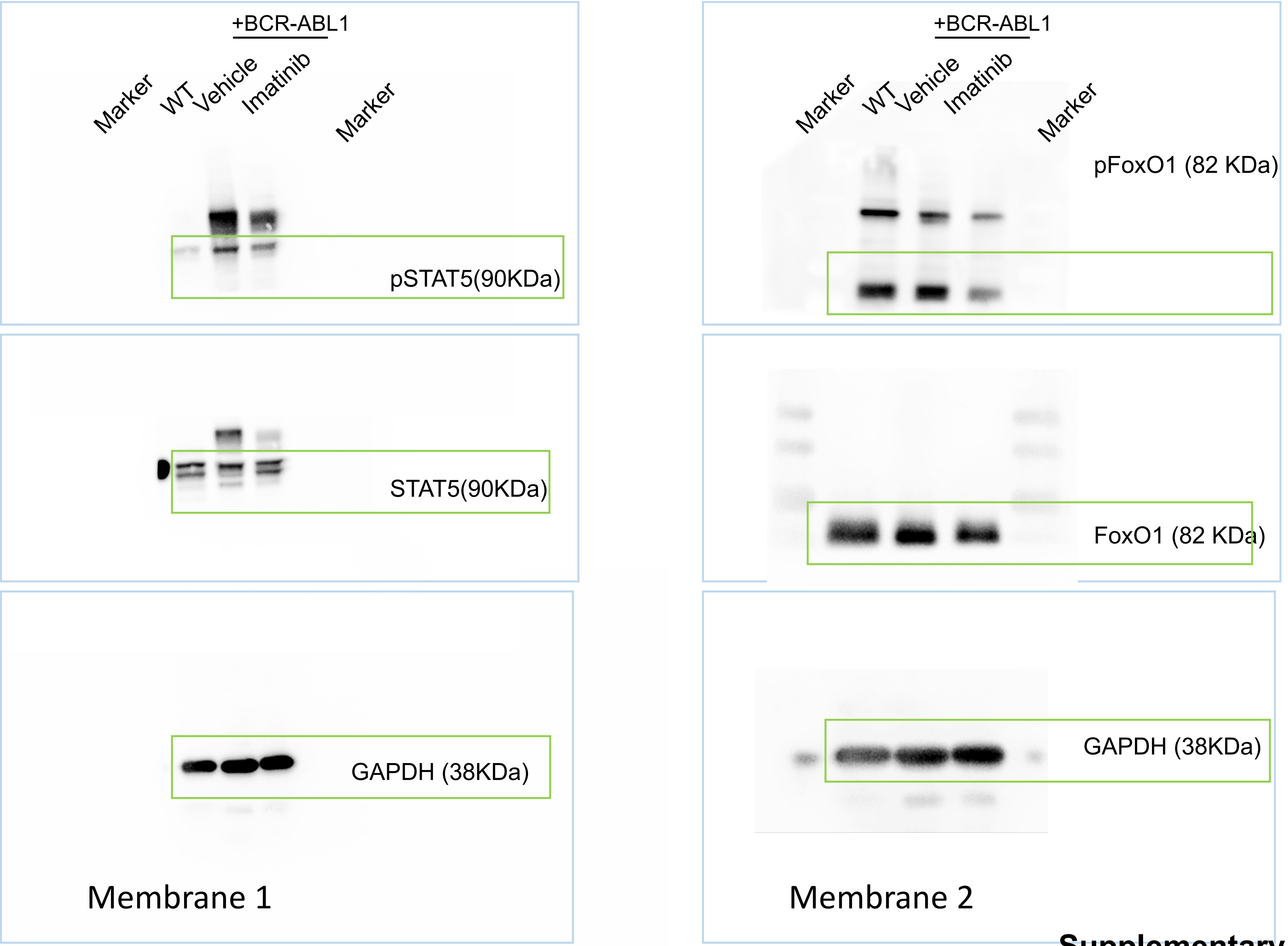

Supplementary Figure 15b

Original Gel Blots for Fig 5b

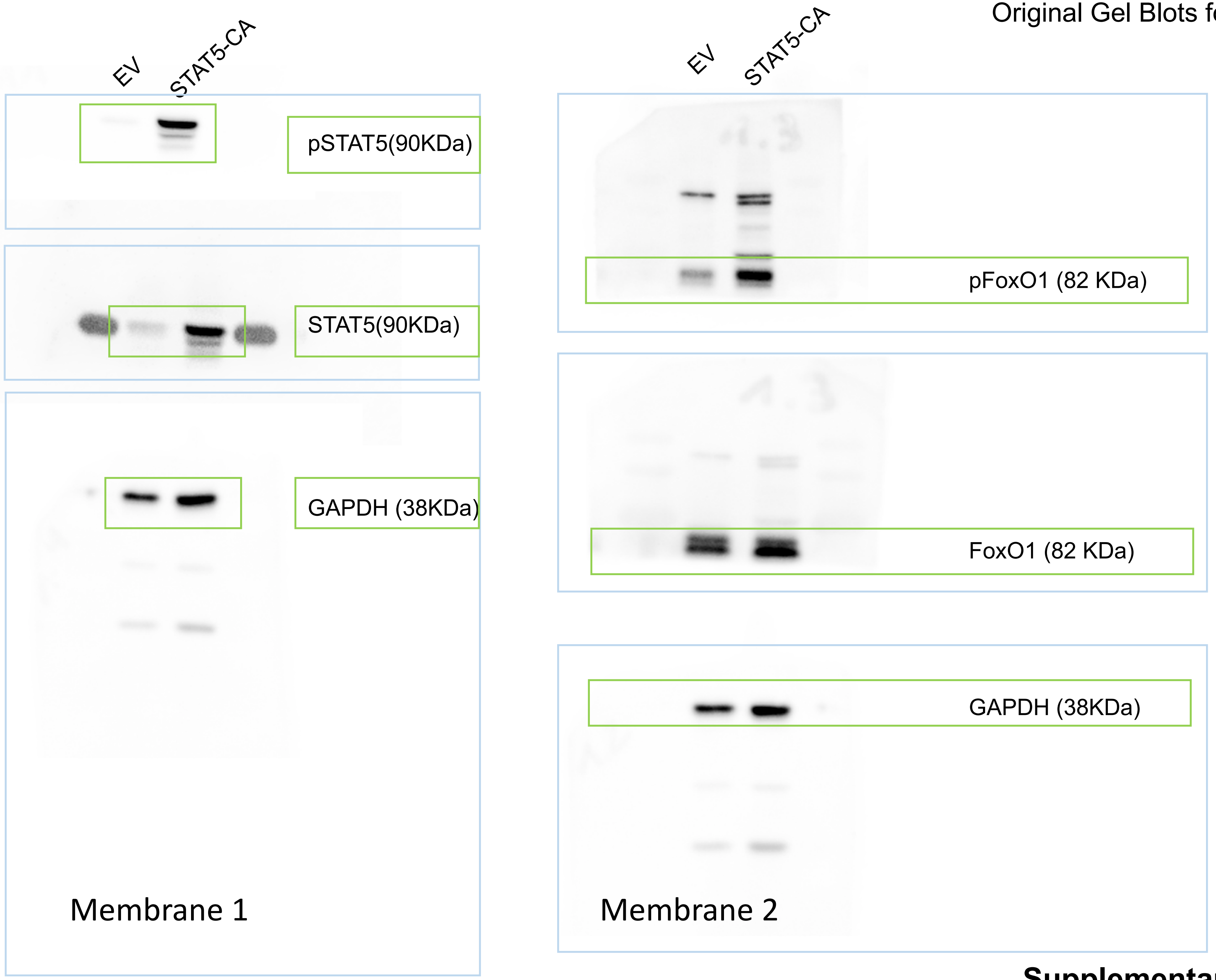

Supplementary Figure 15c

Original Gel Blots for
Supp Fig 8d

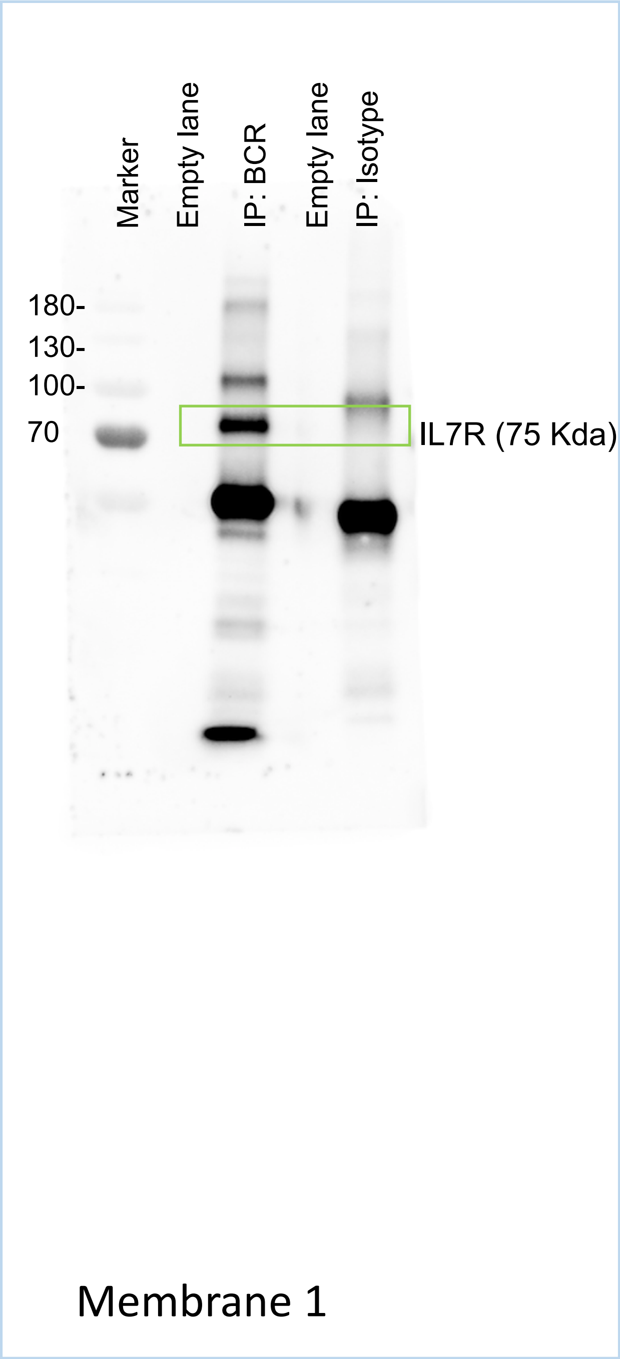

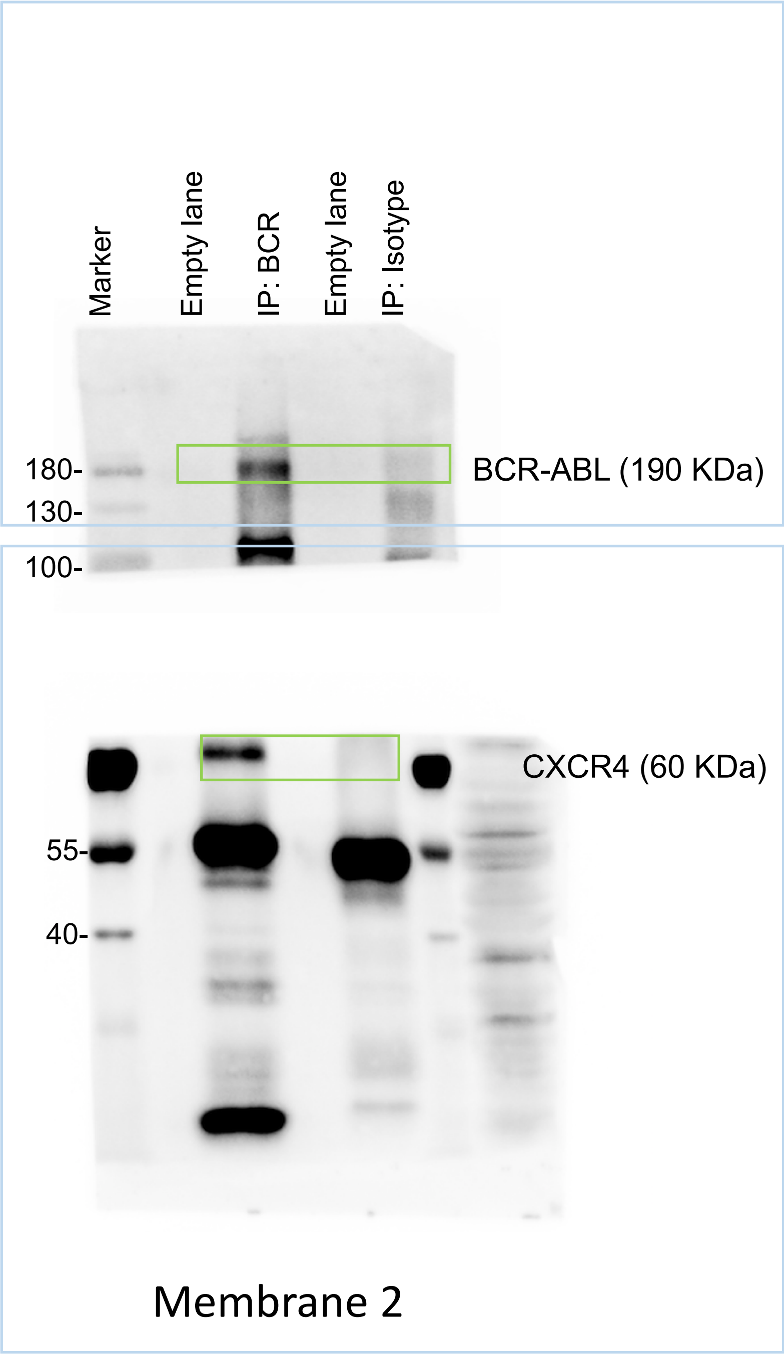

Supplementary Figure 15d

Original Gel Blots for Supp Fig 13c

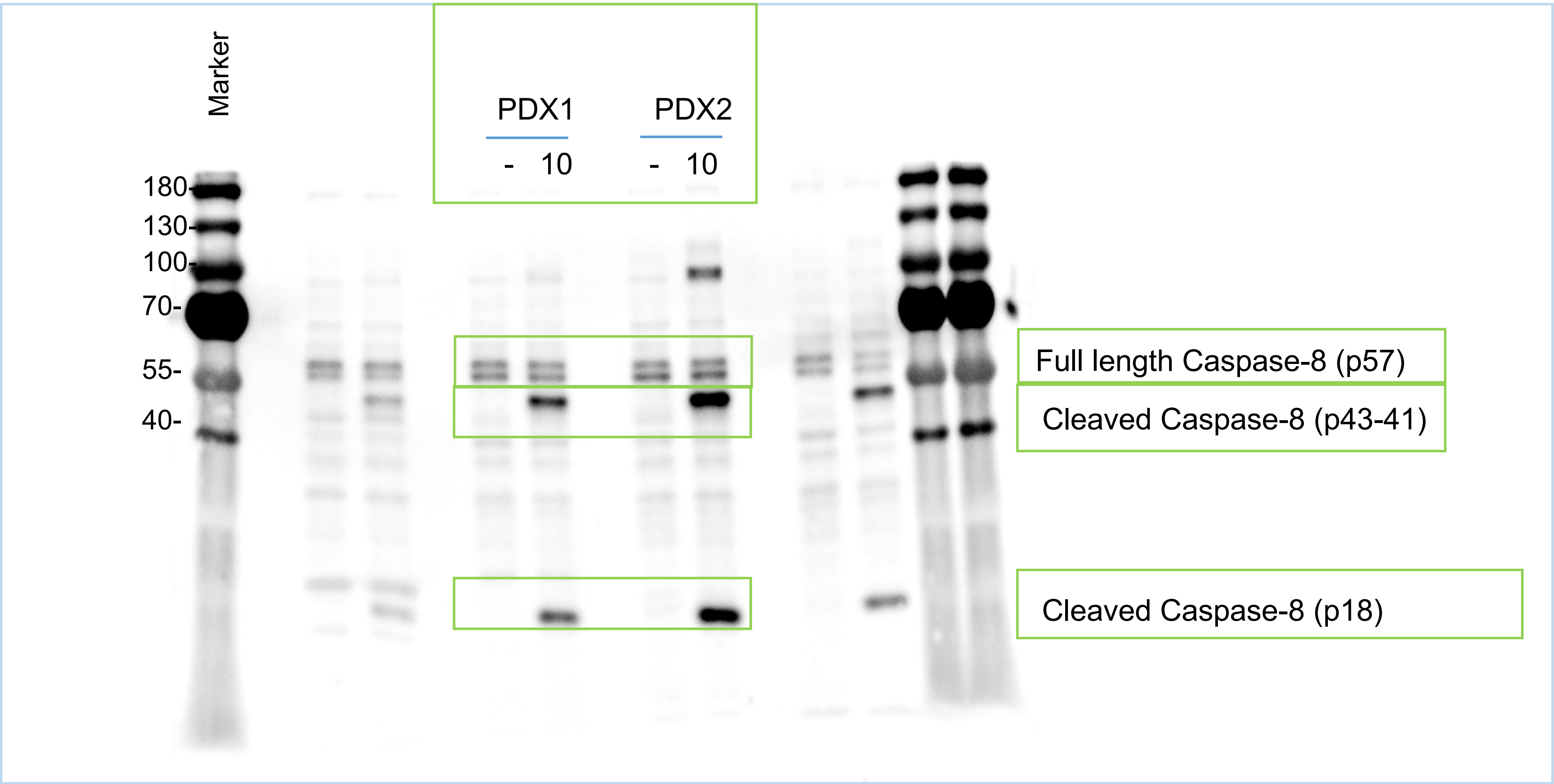

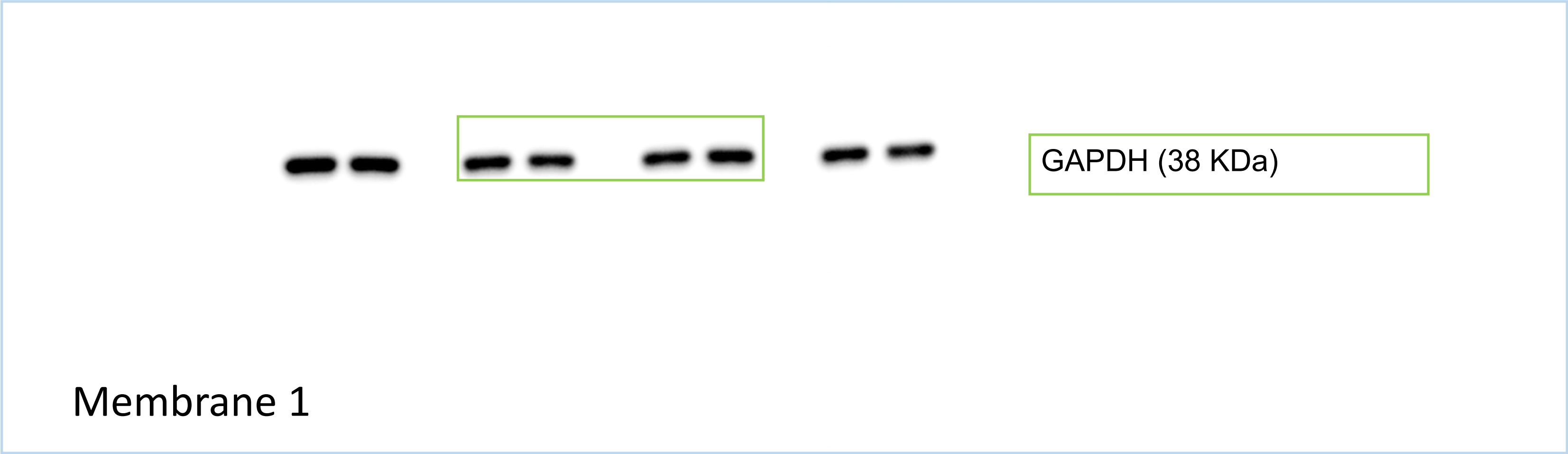

Supplementary Figure 15e

Supplementary Figure 15: Uncropped original gel scans. Original Gel blots for Figure 4a (a), Figure 5a (b), Figure 5b (c), Supplementary Figure 8d (d) and Supplementary Figure 13c (e). Pelase note that in some cases membranes were cut at certain sizes to allow blotting with different antibodies to avoid background resulting from multiple developments on the same membrane.

NAME	SIZE	ES	NES	NOM p-val	FDR q-val	FWER p-val	RANK AT MAX	LEADING EDGE
REACTOME_CYTOKINE_SIGNALING_IN_IMMUNE_SYSTEM	227	0.469654	1.5316948	0.044715445	0.08022534	0.032	1943	tags=21%, list=12%, signal=23%
KEGG_JAK_STAT_SIGNALING_PATHWAY	134	0.5169038	1.5270525	0	0.043434933	0.039	1763	tags=21%, list=11%, signal=23%
REACTOME_IMMUNE_SYSTEM	791	0.36954993	1.5164844	0.022044089	0.029883482	0.039	2733	tags=20%, list=17%, signal=23%
REACTOME_SIGNALING_BY_ILS	102	0.36833644	1.4254694	0.011538462	0.050893776	0.088	1515	tags=12%, list=9%, signal=13%
KEGG_CYTOKINE_CYTOKINE_RECEPTOR_INTERACTION	222	0.4586507	1.1779916	0.15546219	0.24941932	0.509	1585	tags=18%, list=10%, signal=20%
KEGG_HEMATOPOIETIC_CELL_LINEAGE	75	0.37228566	0.98426795	0.4484127	0.4612367	0.787	845	tags=13%, list=5%, signal=14%
KEGG_PRIMARY_IMMUNODEFICIENCY	35	-0.4811019	-1.2607687	0.08213552	0.23853628	0.34	1970	tags=26%, list=12%, signal=29%
REACTOME_IL_7_SIGNALING	11	-0.5914329	-1.2607011	0.22040816	0.119774215	0.341	3015	tags=27%, list=18%, signal=33%

Supplementary Table 1. Statistically significantly upregulated genesets related to IL7R. FDR < 0.25; highlighted in green. A two-sided signal-to-noise metric was used to rank the genes. For a calculated GSEA nominal *p*-values of 0, we present them as $p < 0.001$ (otherwise, exact *p*-values are shown). Multiple hypothesis testing correction is represented by the estimated FDR.

	No.	%	Statistics
**BCR-ABL+ALL**	**68**	**100**	***P***
**Sex***			0.2830
Male	27	44	
Female	34	56	
**Age, years***			0.2720
< 10 years	32	52	
≥ 10 years	29	48	
**Risk group[1]‡**			0.1986
SR	25	41	
IR	25	41	
HR	11	18	
**Prednisone Response[1]***			0.2420
Good	51	83	
Poor	10	17	
**Relapse and Death***			0.2620
No	59	97	
Yes	2	3	

Supplementary Table 2: Clinical parameters of 68 BCR-ABL positive patients at initial diagnosis. [1]SR – standard risk, IR – intermediate risk, HR – high risk. Risk stratification according to minimal residual disease (MRD) risk groups: MRD-SR: TP1+2 negative, MRD-IR: TP1 and/or TP2 <10-3, MRD-HR: TP2 ≥ 10-3. MRD risk group was missing for 2 patients in the CNS pos. Prednisone poor responders were stratified into the HR treatment group.

* Mann-Whitney test, 2-sided *P* value.

‡1-way ANOVA

Supplementary Table 3: Additional information to Boxplots in Supplementary Figure 3.

Information related to Supplementary Figure 3a

IL7R		
	BCR-ABL1 neg (n=1056)	BCR-ABL1 pos (n=167)
Number of values	1056	167
Minimum	6,224	4,516
Maximum	15,93	14,41
Range	9,704	9,892
2.5% Percentile	8,176	6,776
97.5% Percentile	13,6	13,34
Mean	11,32	10,48

CXCR4		
	BCR-ABL1 neg (n=1056)	BCR-ABL1 pos (n=167)
Number of values	1056	167
Minimum	9,384	11
Maximum	17,94	17,1
Range	8,552	6,095
2.5% Percentile	12,7	12,11
97.5% Percentile	16,73	16,73
Mean	14,81	14,53

Information related to Supplementary Figure 3b (http://r2.amc.nl).

**Supplementary Figure 3b**		**IL7R**							**CXCR4**						
	Leukemia type	min	Q25	med	Q75	max	low extreme	high extreme	min	Q25	med	Q75	max	low extreme	high extreme
1	all with hyperdiploid karyotype	6,6	7,62	7,95	8,31	9,33	6,14	9,68	11,87	12,59	12,93	13,28	13,55	n	n
2	all with t(12_21)	6	7,9	8,22	9,16	10,32	4,07	n	11,91	12,59	13,07	13,33	13,73	n	n
3	all with t(1_19)	6,27	8,54	9,39	10,05	11,17	4,68	n	11,8	12,59	12,85	13,11	13,86	11,08	n
4	aml complex aberrant karyotype	2,43	5,15	5,82	6,99	9,09	n	n	11,43	12,18	12,39	12,67	13,41	9,69	13,57
5	aml with inv(16)/t(16_16)	2,5	4,67	5,34	6,12	7,64	1,43	n	10,89	11,62	12,19	12,68	13,09	n	n
6	aml with normal karyotype + other abnormalities	1,57	4,62	5,62	6,65	9,7	0,68	10,65	9,36	11,35	12,16	12,68	13,92	7,69	n
7	aml with t(11q23)/mll	2,07	4,74	5,92	7	9,73	n	n	9,53	11,44	12,26	12,71	13,30	9,22	n
8	aml with t(15_17)	2,39	5,03	5,89	6,79	8,56	2,1	n	11,04	11,81	12,07	12,33	13,10	10,80	13,26
9	aml with t(8_21)	2,68	4,95	5,96	6,78	7,92	n	n	10,24	11,39	11,86	12,68	13,35	n	n
10	c-all/pre-b-all with t(9_22)	3,24	6,17	7,30	8,13	11,06	1,43	11,37	10,95	12,23	12,78	13,08	13,80	10,49	n
11	c-all/pre-b-all without t(9_22)	4,12	6,95	8,00	8,84	10,65	0,48	n	11,72	12,75	13,14	13,43	14,19	9,84	n
12	cll	2,93	5,70	6,74	7,55	10,32	1,14	10,60	12,47	13,20	13,46	13,68	14,41	10,86	14,86
13	cml	2,02	4,58	5,22	6,29	8,28	0,48	n	12,14	12,80	13,03	13,24	13,80	10,85	n
14	mature b-all with t(8_14)	4,38	6,36	7,40	8,04	10,29	n	n	11,74	12,50	12,82	13,01	13,54	11,49	n
15	mds	1,08	5,07	6,40	7,74	10,73	0,76	n	10,69	11,89	12,32	12,69	13,89	10,54	14,12
16	non-leukemia and healthy bone marrow	3,35	6,44	7,65	8,50	10,35	2,72	n	11,67	12,42	12,68	12,91	13,66	11,54	13,77
17	pro-b-all with t(11q23)/mll	5,94	8,01	8,78	9,58	10,62	n	n	11,46	12,55	12,91	13,28	13,85	11,28	n
18	t-all	4,39	7,27	8,25	9,18	11,72	3,14	n	10,54	11,82	12,29	12,67	13,95	10,13	14,05

Antibodies used for flow cytometry									
**Antibody**	**Specificity**	**Host/ Isotype**	**Conjugate**	**Clone**	**Class**	**Supplier**	**Catalog number**	**Lot number**	**Dilution**
CD8	Mouse	Rat/IgG2a	PE	53-6.7	Monoclonal	BD Biosciences	553033	64081	1:100
CD11b	Mouse	Rat/IgG2bk	PE	M1/70	Monoclonal	invitrogen	12-0112-82	1912143	1:400
CD19	Mouse	Rat/IgG2aκ	PerCP-Cy 5.5	1D3	Monoclonal	BD Biosciences	551001	9178619	1:200
B220(CD45)	Mouse/Human	Rat/IgG2aκ	PE-Cy7	RA3-6B2	Monoclonal	Invitrogen	25-0452-82	2008222	1:200
CD127 (IL7R)	Mouse/Human	Rat/IgG2aκ	APC	D7715A7	Monoclonal	BD Biosciences	564175	7215841	1:100
CD127(IL7R)	Mouse/Human	Rat/IgG2aκ	eFluor 660	A7R34	Monoclonal	Invitrogen	50-1271-82	4335207	1:100
CXCR4	Mouse	Rat/IgG2bκ	Alxa Fluor 647	L276F12	Monoclonal	BioLegend	146504	B250543	1:60
CXCR4	Mouse	Rat/IgG2bκ	BV421	L276F12	Monoclonal	BioLegend	146511	B241213	1:100
Kappa	Mouse	Goat/IgG	PE	-	polyclonal	SouthernBiotech	1050-09	C0916-RG37B	1:100
IgM	Mouse	Goat/IgG	Cy5	-	Polyclonal	Jackson ImmunoResearch	115-175-075	Unknown	1:300
IgM	Mouse	Goat/IgG2ak	eFluor450	eb121-15F9	Monoclonal	eBioscience	48-5890-82	2082929	1:300

IgM	Mouse	Rat/IgG1	APC	1B4B1	Monoclonal	SouthernBiotech	1140-11	E5802-M523X	1:100
FOXO1	Mouse/Human	Rabbit/IgG	unlabeled	C29H4	Monoclonal	Cell signaling	2880S	11	1:100
Anti-rabbit IgG (H+L), F(ab')2 Fragment	Anti Rabbit IgG Fab2	Goat	AF647	-	Polyclonal	Cell signaling	4414	16	1:200
hCD19	Human	Mouse/IgG1k	PE	HIB19	Monoclonal	BioLegend	302208	B273506	1:200
mCD45	Mouse	Rat/IgG2b	APC	I3/2.3	Monoclonal	BioLegend	147708	B237012	1:100
hCD45	Human	Mouse/IgG1K	FITC	HI30	Monoclonal	BioLegend	304006	B234201	1:100
**Antibodies used for western blot**									
**Antibody**	**Specificity**	**Host/Isotype**	**Conjugate**	**Clone**	**Class**	**Supplier**	**Catalog number**	**Lot number**	**Dilution**
FOXO1	Mouse/Human	Rabbit/IgG	unlabeled	C29H4	Monoclonal	Cell signaling	2880	11	1:1000
pFoxO1		Rabbit	unlabeled	E1F7T	Monoclonal	Cell signaling	84192	1	1:1000
pAKT (S473)	Mouse/Human	Rabbit/IgG	unlabeled	D9E	Monoclonal	Cell signaling	4060	24	1:1000
pSTAT5	Mouse/Human	Rabbit/IgG	unlabeled	C71E5	Monoclonal	Cell signaling	9314	22	1:1000
STAT5	Mouse/Human	Rabbit/IgG	unlabeled	D3N2B	Monoclonal	Cell signaling	9363	3	1:1000
JAK1	Mouse/Human	Rabbit/IgG	unlabeled	64G	Monoclonal	Cell signaling	3332	6	1:1000
JAK2	Mouse/Human	Rabbit/IgG	unlabeled	D2E12	Monoclonal	Cell signaling	3230	11	1:1000
JAK3	Mouse/Human	Rabbit/IgG	unlabeled	D7B12	Monoclonal	Cell signaling	8863	Unknown	1:1000
pJAK1 (Y1022/1023)	Mouse/Human	Rabbit	unlabeled	-	Polyclonal	Cell signaling	3331	5	1:1000

pJAK2 (Y1007/1008)	Mouse/Human	Rabbit	unlabeled		Polyclonal	Cell signaling	3771	10	1:1000
pJAK3 (Y980)	Mouse/Human	Rabbit	unlabeled	D44E3	Monoclonal	Cell signaling	5031	7	1:1000
GAPDH		Rabbit	unlabeled	14C10	Monoclonal	Cell signaling	2118	10	1:1000
Anti-rabbit IgG	Rabbit	Goat	HRP-linked	-	Polyclonal	Cell signaling	7074	27	1:1000

Supplementary Table 4: List of all antibodies used in the Manuscript.

Supplementary Methods

Ruxolitinib, imatinib and antibody treatment *in vivo*

NSG mice were injected with 1×10^6 SUP-B15 BCR-ABL positive ALL cells/animal. 60 mg/kg of ruxolitinib (LC LAboratories) or 40 mg/kg of imatinib (LC Laboratories) or combination of both inhibitors were administered orally 5 days a week. Mice were sacrificed when they showed signs of leukemia. Mice were randomly allocated into each treatment group and no blinding was used.

Cell cycle analysis

For cell cycle analyses, Click-iT® EdU Alexa Fluor® 647 Imaging Kit (Invitrogen) or BrdU Flow Kit (BD Biosciences) were used.

Colony Formation Assay

1×10^5 $CXCR4^{fl/fl}$ mb1 Cre-ERT2 cells transformed with pMIG-BCR ABL1 were treated with either ethanol or tamoxifen and then used for colony formation assay as described previously[1].

Chemotaxis

Chemotaxis assay was performed as described[2]. 5×10^5 cells were seeded on the top chamber of a transwell culture insert (Corning) and allowed to migrate toward media containing 100 ng/ml CXCL12 (ImmunoTools) for 16 h. The cell number in the lower chamber was determined with hematocytometer.

Immunoprecipitation

SUP-B15 Ph+ ALL cells were lysed and an Immunoprecipitation (IP; Dynabeads™ Protein A Immunoprecipitation kit; Thermoscientific) was performed using an antibody against the breakpoint cluster region protein (BCR; SantaCruz), or IgG2a (isotype; Southern Biotech). The proteins from the IP were used for western blotting to detect the presence of IL7R and CXCR4 proteins (R&D, and Invitrogen, respectively).

Statistical analysis

Statistical tests are indicated in the figure legends. Results were analyzed for statistical significance with GraphPad Prism 6 software or SPSS. A p value of <0.0500 was considered significant. *In vitro* panels are representative of at least 3 independent experiments.

RNA Sequencing data analysis

RNA-seq analyses were performed using the following softwares: the base calling was performed by using BCL2Fastq pipeline (v. 0.3.0) and bcl2fastq (v. 2.17.1.14). The quality of the raw paired-end reads from the RNA-Seq dataset was assessed using the programm FastQC (v. 0.11.5)[3]. After adapter clipping, quality trimming and length filtering was performed using the program Trimmomatic (v.0.36)[4], the high-quality paired-end reads were mapped in a paired-end aware, strand-specific manner to the Ensembl mouse reference genome (GRCm38.dna.chr) using the splice-aware mapper

TopHat (v. 2.1.1)[5] with the aligner Bowtie2 (v. 2.3.2.)[6]. FeatureCounts[7] was used to count uniquely mapped read pairs in a strand-specific manner using the Ensembl mouse gene annotation (GRCm38.89.chr.gtf) to create a raw read count table which was then read into the R statistical computing environment (https://www.R-project.org/). The edgeR Bioconductor package (v. 3.18.1)[8] was then used to normalize the filtered read counts (TMM normalization)[9] and to test for differentially expressed genes using the glmTreat function (FDR<0.05; fold change > 1.5). Stand alone GSEA package[10] and Molecular Signatures Database (MSigDB; v. 6.2)[11] were used for gene set enrichment analysis. PCA, Differential expression analysis and additional statistical tests related to RNA-seq were performed using R and bioconductor packages[12-16].

Supplementary References

1. Miller, C.L. & Lai, B. Human and mouse hematopoietic colony-forming cell assays. *Methods Mol Biol* **290**, 71-89 (2005).
2. Calpe, E.*, et al.* ZAP-70 enhances migration of malignant B lymphocytes toward CCL21 by inducing CCR7 expression via IgM-ERK1/2 activation. *Blood* **118**, 4401-4410 (2011).
3. Andrews, S. FastQC: a quality control tool for high throughput sequence data. (2010).
4. Bolger, A.M., Lohse, M. & Usadel, B. Trimmomatic: a flexible trimmer for Illumina sequence data. *Bioinformatics* **30**, 2114-2120 (2014).
5. Kim, D.*, et al.* TopHat2: accurate alignment of transcriptomes in the presence of insertions, deletions and gene fusions. *Genome Biol* **14**, R36 (2013).
6. Langmead, B. & Salzberg, S.L. Fast gapped-read alignment with Bowtie 2. *Nat Methods* **9**, 357-359 (2012).
7. Liao, Y., Smyth, G.K. & Shi, W. featureCounts: an efficient general purpose program for assigning sequence reads to genomic features. *Bioinformatics* **30**, 923-930 (2014).
8. Robinson, M.D., McCarthy, D.J. & Smyth, G.K. edgeR: a Bioconductor package for differential expression analysis of digital gene expression data. *Bioinformatics* **26**, 139-140 (2010).
9. Robinson, M.D. & Oshlack, A. A scaling normalization method for differential expression analysis of RNA-seq data. *Genome Biol* **11**, R25 (2010).
10. Subramanian, A.*, et al.* Gene set enrichment analysis: a knowledge-based approach for interpreting genome-wide expression profiles. *Proc Natl Acad Sci U S A* **102**, 15545-15550 (2005).
11. Liberzon, A.*, et al.* Molecular signatures database (MSigDB) 3.0. *Bioinformatics* **27**, 1739-1740 (2011).
12. R Core Team. R: A language and environment for statistical computing. (R Foundation for Statistical Computing, Vienna, Austria. , 2015).
13. Huber, W.*, et al.* Orchestrating high-throughput genomic analysis with Bioconductor. *Nat Methods* **12**, 115-121 (2015).
14. Ploner, A. Heatmaps with row and/or column covariates and colored clusters.
15. Warnes, G.R.*, et al.* gplots: Various R Programming Tools for Plotting Data.
16. Adler, D.*, et al.* RGL - 3D visualization device system for R using OpenGL.
