# Supplementary material for: Synergism between IL7R and CXCR4 drives BCR-ABL induced transformation in Philadelphia chromosome-positive acute lymphoblastic leukemia
Source: Nat Commun. 2020 Jun 24;11:3194. doi: 10.1038/s41467-020-16927-w (PMC7314847; doi:10.1038/s41467-020-16927-w)
Supplement: Supplementary file 3 — Description of Additional Supplementary Files [file 41467_2020_16927_MOESM3_ESM.docx]

**Description of Additional Supplementary Files**

**File Name: Supplementary Data 1**

**Description: Gene expression and Gene Ontology (GO) Analyses.** Included is list of all genes differentially expressed between pre-B cells transduced with empty vector or transformed with BCR-ABL1 as well as lists for selected GO, namely GO-0016477_Cell_Migration, GO-0007159_Leukocyte_cell-cell adhesion, GO-0050900_Leukocyte Cell Migration, GO-0046649_Lymphocyte_Activation, GO-0046651_Lymphocyte_Proliferation, and GO-0030098_Lymphocyte_Differentiation. The glmTreat function (FDR<0.05; fold change threshold > 1.5) from the edgeR Bioconductor package was used to conduct a modified likelihood ratio test (LRT) against the fold-change threshold in order to identify differentially expressed (DE) genes. To test for over-representation of GO terms, R’s goana function was employed to conduct overlap tests for the up- and down-regulated DE genes separately (one-sided hypergeometric tests equivalent to Fisher's exact test; FDR<0.05).
