# Supplementary material for: Synergism between IL7R and CXCR4 drives BCR-ABL induced transformation in Philadelphia chromosome-positive acute lymphoblastic leukemia
Source: Nat Commun. 2020 Jun 24;11:3194. doi: 10.1038/s41467-020-16927-w (PMC7314847; doi:10.1038/s41467-020-16927-w)
Supplement: Supplementary file 5 — Reporting Summary [file 41467_2020_16927_MOESM5_ESM.pdf]

nature research

Corresponding author(s): Hassan Jumaa

Last updated by author(s): May 22, 2020

Reporting Summary

Nature Research wishes to improve the reproducibility of the work that we publish. This form provides structure for consistency and transparency in reporting. For further information on Nature Research policies, see Authors & Referees and the Editorial Policy Checklist .

Statistics

For all statistical analyses, confirm that the following items are present in the figure legend, table legend, main text, or Methods section.

n/a	Confirmed	
☐	☒	The exact sample size (*n*) for each experimental group/condition, given as a discrete number and unit of measurement
☐	☒	A statement on whether measurements were taken from distinct samples or whether the same sample was measured repeatedly
☐	☒	The statistical test(s) used AND whether they are one- or two-sided<br>*Only common tests should be described solely by name; describe more complex techniques in the Methods section.*
☐	☒	A description of all covariates tested
☐	☒	A description of any assumptions or corrections, such as tests of normality and adjustment for multiple comparisons
☐	☒	A full description of the statistical parameters including central tendency (e.g. means) or other basic estimates (e.g. regression coefficient) AND variation (e.g. standard deviation) or associated estimates of uncertainty (e.g. confidence intervals)
☐	☒	For null hypothesis testing, the test statistic (e.g. *F*, *t*, *r*) with confidence intervals, effect sizes, degrees of freedom and *P* value noted<br>*Give P values as exact values whenever suitable.*
☒	☐	For Bayesian analysis, information on the choice of priors and Markov chain Monte Carlo settings
☒	☐	For hierarchical and complex designs, identification of the appropriate level for tests and full reporting of outcomes
☒	☐	Estimates of effect sizes (e.g. Cohen's *d*, Pearson's *r*), indicating how they were calculated

Our web collection on statistics for biologists contains articles on many of the points above.

Software and code

Policy information about availability of computer code

Data collection	No software was used for data collection
Data analysis	Image Analysis (PLA): Image J (v. 1.x) and BlobFinder software (v. 3.2)<br>FACS Analysis: FLowJo (v. 10.4)<br>Statistical analyses: GraphPad Prism(v. 8.3.0) or SPSS (v. 24)<br>RNA-seq analyses were performed using the following softwares: the base calling was performed by using BCL2Fastq pipeline (v. 0.3.0) and bcl2fastq (v. 2.17.1.14). PCA, Differential expression analysis and additional statistical tests related to RNA-seq were performed using R and bioconductor packages. The quality of the raw paired-end reads from the RNA-Seq dataset was assessed using the programm FastQC (v. 0.11.5) (Andrews 2010) . After adapter clipping, quality trimming and length filtering was performed using the program Trimmomatic (v.0.36) (Bolger et. al.,2014) , the high-quality paired-end reads were mapped in a paired-end aware, strand-specific manner to the Ensembl mouse reference genome (GRCm38.dna.chr) using the splice-aware mapper TopHat (v. 2.1.1) (Kim et. al. 2013) with the aligner Bowtie2 (v. 2.3.2.) (Langmead et al. 2012). FeatureCounts (Liao et al. 2014) was used to to count uniquely mapped read pairs in a strand-specific manner using the Ensembl mouse gene annotation (GRCm38.89.chr.gtf) to create a raw read count table which was then read into the R statistical computing environment (https://www.R-project.org/). The edgeR Bioconductor package (v. 3.18.1) (Robinson et al. 2010) was then used to normalize the filtered read counts (TMM normalization) and to test for differentially expressed genes using the glmTreat function (FDR<0.05; fold change > 1.5). Stand alone GSEA package (Subramanian et al. 2005) and Molecular Signatures Database (MSigDB; v. 6.2) (Liberzon et al. 2011) were used for gene set enrichment analysis"<br>The scripts used for analysis and figure generation are available at https://github.com/medhaniea/pca-and-heatmap.

For manuscripts utilizing custom algorithms or software that are central to the research but not yet described in published literature, software must be made available to editors/reviewers. We strongly encourage code deposition in a community repository (e.g. GitHub). See the Nature Research guidelines for submitting code & software for further information.

Data

Policy information about availability of data

All manuscripts must include a data availability statement. This statement should provide the following information, where applicable:

- Accession codes, unique identifiers, or web links for publicly available datasets
- A list of figures that have associated raw data
- A description of any restrictions on data availability

A data availability statement, containing accession code, unique identifiers as well as web link for publicly available datasets and a list of all associated figures or data, is included in the manuscript.

Field-specific reporting

Please select the one below that is the best fit for your research. If you are not sure, read the appropriate sections before making your selection.

☒ Life sciences ☐ Behavioural & social sciences ☐ Ecological, evolutionary & environmental sciences

For a reference copy of the document with all sections, see nature.com/documents/nr-reporting-summary-flat.pdf

Life sciences study design

All studies must disclose on these points even when the disclosure is negative.

Sample size	No sample size calculation was applied in this study. Experiments were performed independently for at least 3 times in an unbiased manner. Animal and patient sample size was determined by experimental feasibility and sample availability to demonstrate certain results.
Data exclusions	No data was purposely omitted for the purpose of data analyses.
Replication	All in vitro studies included biological replications (three biological replicates at least) and statistics were indicated in the legends. Experiments were performed by at least two researchers to ensure reproducibility. All attempts of replication were successful. In vivo validation was conducted by an independent research team.
Randomization	Mice, with comparable weights, were randomly allocated into each treatment group. For in vitro experiments, samples were allocated into experimental groups by the confirmed genetic modification of the cell line (e.g. Tamoxofen-induced deletion, STAT5-CA protein overexpression) and/or culturing conditions (e.g. veihcle or treatment).
Blinding	Investigators were blinded to group allocation and data analysis.

Reporting for specific materials, systems and methods

We require information from authors about some types of materials, experimental systems and methods used in many studies. Here, indicate whether each material, system or method listed is relevant to your study. If you are not sure if a list item applies to your research, read the appropriate section before selecting a response.

Materials & experimental systems

n/a	Involved in the study	
☐	☒	Antibodies
☐	☒	Eukaryotic cell lines
☒	☐	Palaeontology
☐	☒	Animals and other organisms
☐	☒	Human research participants
☐	☒	Clinical data

Methods

n/a	Involved in the study	
☒	☐	ChIP-seq
☐	☒	Flow cytometry
☒	☐	MRI-based neuroimaging

Antibodies

Antibodies used

Antibodies used for flow cytometry
Antibody Specificity Host/ Isotype Conjugate Clone Class Supplier Catalog number Lot number Dilution
CD8 Mouse Rat/IgG2a PE 53-6.7 Monoclonal BD Biosciences 553033 64081 1:100
CD11b Mouse Rat/IgG2bk PE M1/70 Monoclonal invitrogen 12-0112-82 1912143 1:400
CD19 Mouse Rat/IgG2aκ PerCP-Cy 5.5 1D3 Monoclonal BD Biosciences 551001 9178619 1:200
B220(CD45) Mouse/Human Rat/IgG2aκ PE-Cy7 RA3-6B2 Monoclonal Invitrogen 25-0452-82 2008222 1:200
CD127 (IL7R) Mouse/Human Rat/IgG2aκ APC D7715A7 Monoclonal BD Biosciences 564175 7215841 1:100
CD127(IL7R) Mouse/Human Rat/IgG2aκ eFluor 660 A7R34 Monoclonal Invitrogen 50-1271-82 4335207 1:100

CXCR4 Mouse Rat/IgG2bκ Alxa Fluor 647 L276F12 Monoclonal BioLegend 146504 B250543 1:60
CXCR4 Mouse Rat/IgG2bκ BV421 L276F12 Monoclonal BioLegend 146511 B241213 1:100
Kappa Mouse Goat/IgG PE - polyclonal SouthernBiotech 1050-09 C0916-RG37B 1:100
IgM Mouse Goat/IgG Cy5 - Polyclonal Jackson ImmunoResearch 115-175-075 Unknown 1:300
IgM Mouse Goat/IgG2ak eFluor450 eb121-15F9 Monoclonal eBioscience 48-5890-82 2082929 1:300
IgM Mouse Rat/IgG1 APC 1B4B1 Monoclonal SouthernBiotech 1140-11 E5802-M523X 1:100
FOXO1 Mouse/Human Rabbit/IgG unlabeled C29H4 Monoclonal Cell signaling 2880S 11 1:100
Anti-rabbit IgG (H+L), F(ab')2 Fragment Anti Rabbit IgG Fab2 Goat AF647 - Polyclonal Cell signaling 4414 16 1:200
hCD19 Human Mouse/IgG1k PE HIB19 Monoclonal BioLegend 302208 B273506 1:200
mCD45 Mouse Rat/IgG2b APC I3/2.3 Monoclonal BioLegend 147708 B237012 1:100
hCD45 Human Mouse/IgG1K FITC HI30 Monoclonal BioLegend 304006 B234201 1:100

Antibodies used for western blot
Antibody Specificity Host/Isotype Conjugate Clone Class Supplier Catalog number Lot number Dilution
FOXO1 Mouse/Human Rabbit/IgG unlabeled C29H4 Monoclonal Cell signaling 2880 11 1:1000
pFoxO1 Rabbit unlabeled E1F7T Monoclonal Cell signaling 84192 1 1:1000
pAKT (S473) Mouse/Human Rabbit/IgG unlabeled D9E Monoclonal Cell signaling 4060 24 1:1000
pSTAT5 Mouse/Human Rabbit/IgG unlabeled C71E5 Monoclonal Cell signaling 9314 22 1:1000
STAT5 Mouse/Human Rabbit/IgG unlabeled D3N2B Monoclonal Cell signaling 9363 3 1:1000
JAK1 Mouse/Human Rabbit/IgG unlabeled 64G Monoclonal Cell signaling 3332 6 1:1000
JAK2 Mouse/Human Rabbit/IgG unlabeled D2E12 Monoclonal Cell signaling 3230 11 1:1000
JAK3 Mouse/Human Rabbit/IgG unlabeled D7B12 Monoclonal Cell signaling 8863 Unknown 1:1000
pJAK1(Y1022/1023) Mouse/Human Rabbit unlabeled - Polyclonal Cell signaling 3331 5 1:1000
pJAK2(Y1007/1008) Mouse/Human Rabbit unlabeled Polyclonal Cell signaling 3771 10 1:1000
pJAK3(Y980) Mouse/Human Rabbit unlabeled D44E3 Monoclonal Cell signaling 5031 7 1:1000
GAPDH Rabbit unlabeled 14C10 Monoclonal Cell signaling 2118 10 1:1000
Anti-rabbit IgG Rabbit Goat HRP-linked - Polyclonal Cell signaling 7074 27 1:1000

Validation

All primary antibodies were validated commercially and reviewed by peers. Each primary antibody data provided in the manuscript has been validated for the species and application on the manufacturer's website. Detailed information are provided in supplementary table 4.

Eukaryotic cell lines

Policy information about cell lines

Cell line source(s)

697, SUP-B15, and TOM-1 cell lines were obtained from DSMZ.

Authentication

Cell lines were authenticated using FACS (markers for BCP-ALL such as hCD19, hCD45) and RT-PCR was used to confirm the chromosomal translocation for BCR-ABL+ phenotype

Mycoplasma contamination

All cell lines were tested negative for mycoplasma contamination

Commonly misidentified lines
(See ICLAC register)

No commonly misidentified lines were used in this study

Animals and other organisms

Policy information about studies involving animals; ARRIVE guidelines recommended for reporting animal research

Laboratory animals

WT (B6), IL7Ra KO, IL7Rafl/fl, CXCR4fl/fl, FOXO1fl/fl, NOD-SCID, and NSG mice. Mice used were female and of 8-12 weeks old.

Wild animals

No wild animals were used in this study

Field-collected samples

No field-collected samples were used in this study

Ethics oversight

NSG mice housing, breeding, and surgical procedures were approved by the governmental animal care and use committees in Schleswig-Holstein (Ministerium für Energiewende, Landwirtschaft, Umwelt, Natur und Digitalisierung).
All other mice housing, breeding, and surgical procedures were approved by the governmental institutions of Baden-Württemberg (Regierungspräsidium Tübingen).

Note that full information on the approval of the study protocol must also be provided in the manuscript.

Human research participants

Policy information about studies involving human research participants

Population characteristics	A detailed description of population characteristics is provided in Supplementary table 3.
Recruitment	Patients were consecutively recruited into the clinical trials mentioned in the methods section. Patients for the analyses performed in this manuscript were selected according to BCR-ABL positivity. All patients with BCR-ABL positivity were analyzed, there is no selection bias impacting the results.
Ethics oversight	The EsPhALL 2004 and 2010 and ALL Berlin-Frankfurt-Münster (BFM) 2000 protocols were approved by the Ethical committee of the "Medizinische Hochschule Hannover".

Note that full information on the approval of the study protocol must also be provided in the manuscript.

Clinical data

Policy information about clinical studies

All manuscripts should comply with the ICMJEguidelines for publication of clinical research and a completedCONSORT checklist must be included with all submissions.

Clinical trial registration	NCT00287105 and NCT00430118
Study protocol	Full trial protocol can be accessed: https://clinicaltrials.gov/ct2/show/NCT00287105 and https://clinicaltrials.gov/ct2/show/NCT00430118?term=NCT00430118&draw=2&rank=1
Data collection	1-NCT00287105: Recent advances in treatment have increased the cure of childhood ALL to 75% or better. However, attempts to improve results for resistant subtypes of ALL, such as Ph+ ALL, have been largely unsuccessful. Imatinib, an inhibitor of protein-tyrosine kinases, is currently being tested in several phase I, II and III trials covering most Chronic Myeloid Leukemia patient populations and patients with overtly relapsed or refractory Ph+ALL. Pediatric patients with Ph+ALL will receive Imatinib, added to intensive, post-induction BFM-type chemotherapy. The endpoint will be the evaluation on the long-term clinical outcome, in particular on the Disease Free Survival (DFS).<br>-Study Design:<br>Study Type : Interventional (Clinical Trial)<br>Actual Enrollment : 49 participants<br>Allocation: Non-Randomized<br>Intervention Model: Single Group Assignment<br>Masking: None (Open Label)<br>Primary Purpose: Treatment<br>Official Title: An Open-label, Phase II Study to Explore the Safety and Efficacy of Imatinib With Chemotherapy in Pediatric Patients With Ph+ / BCR-ABL+ Acute Lymphoblastic Leukemia (Ph+ALL)<br>Actual Study Start Date : December 2005<br>Actual Primary Completion Date : March 30, 2016<br>Actual Study Completion Date : March 3, 2017<br>-Locations: please refer to https://clinicaltrials.gov/ct2/show/NCT00287105 for detailed recruitment locations<br><br>2-NCT00430118: Drugs used in chemotherapy work in different ways to stop the growth of cancer cells, either by killing the cells or by stopping them from dividing. Giving more than one drug (combination chemotherapy) may kill more cancer cells. It is not yet known which combination chemotherapy regimen is more effective in treating young patients with acute lymphoblastic leukemia.<br>-PURPOSE: Thisphase III trial is studying several different combination chemotherapy regimens to compare how well they work in treating young patients with acute lymphoblastic leukemia.<br>-Study Design:<br>Study Type : Interventional (Clinical Trial)<br>Actual Enrollment : 4559 participants<br>Allocation: Randomized<br>Intervention Model: Factorial Assignment<br>Masking: None (Open Label)<br>Primary Purpose: Treatment<br>Official Title: ALL-BFM 2000 Multi-Center Study for the Treatment of Children and Adolescents With Acute Lymphoblastic Leukemia<br>Study Start Date : July 2000<br>Actual Primary Completion Date : January 2012<br>Actual Study Completion Date : January 2012<br>--Locations: please refer to https://clinicaltrials.gov/ct2/show/NCT00430118?term=NCT00430118&draw=2&rank=1 for detailed recruitment locations
Outcomes	1-NCT00287105:<br>-Primary Outcome Measures: Disease free survival (DFS). DFS will be calculated as the time from inclusion to either one of the following events: relapse, death in CCR, second malignancies. [Time Frame: 2 years]<br>-Secondary Outcome Measures :

a- Compare long term outcome between patients treated by BFM-chemotherapy and patient undergoing more intensive chemotherapy (protocole COGAALL0031 : Children Oncology Group-USA). [Time Frame: 2 years]
b- Long-term clinical outcome : Disease free survival (DFS), Event-Free Survival (EFS) and Overall Survival (OS) in each risk groups. [Time Frame: 2 years]
c-Pattern of molecular response (MRD) [Time Frame: 5 time points between S4 and S22]
d-Conversion rate to CR in patients resistant to the first part of the induction phase of chemotherapy included in the Poor-risk group. [Time Frame: 2 years]

2-NCT00430118:
Primary Outcome Measures :
a-Efficacy of dexamethasone vs prednisone during the induction phase [Time Frame: End of Trial]
b- Event-free survival (EFS) and overall survival after initial remission in intermediate-risk and high-risk patients [Time Frame: End of Trial]
c-Safety and efficacy of treatment reduction during reintensification in standard-risk patients [Time Frame: End of Trial]
d-EFS after second delayed reintensification in intermediate-risk patients [Time Frame: End of Trial]
e-Outcome after extended reintensification therapy in high-risk patients [Time Frame: End of Trial]

Flow Cytometry

Plots

Confirm that:

- [x] The axis labels state the marker and fluorochrome used (e.g. CD4-FITC).
- [x] The axis scales are clearly visible. Include numbers along axes only for bottom left plot of group (a 'group' is an analysis of identical markers).
- [x] All plots are contour plots with outliers or pseudocolor plots.
- [x] A numerical value for number of cells or percentage (with statistics) is provided.

Methodology

Sample preparation	Samples isolated from Spleen or BM: Mice were sacrificed and specific lymphatic organs (bone marrow and spleen) were isolated and transferred into 5 ml of PBS buffer and single cell suspension was prepared. The cells were centrifuged at 1200 rpm 4°C for 5 min then resuspended in RBC lysis buffer for 3-10 min in ice. Cells were then washed with PBS, were passed through a 40 µm cell strainer and resuspended in FACS buffer. Total cell number was enumerated and dead cells were excluded by trypan blue stain. Otherwise, cells were collected directly from cell culture, washed with PBS and processed as described above.<br>Extracellular staining: 1x106 cells were transferred into a FACS tube and used for staining. The samples were centrifuged and the supernatant was removed, and the cell pellets were resuspended with 25 µl of fluorescent antibody dilution mix. The tubes were incubated on ice covered from light for 20 min. The cells were washed with 0.7-1.0 ml of FACS buffer, and the cell pellet was resuspended in 100-300 µl of FACS buffer. The tubes were kept on ice covered from light before FACS analysis (not later than 2-3 hr).<br>Intracellular staining, at least 1-2x106 cells were used. Surface staining was performed first, when needed, and then the cells were washed once with 1xPBS. Intracellular FACS staining was done using Fix and perm cell permeabilization kit (ADG) following manufacturer's instructions. After fixation 10 min at RT, cells were washed with 500 µl 1x PBS and then incubated with the primary antibody diluted in the kit-accompanied diluent for 12-20 min at RT. The cells were then washed twice with 500 µl freshly made saponin buffer (0.5% saponin, 0.5% BSA and 0.02% NaN3 in PBS). When required, labeled secondary antibody was used and the staining procedure mentioned before was repeated.
Instrument	FACS Canto II Cytometer (BD Biosciences) was used for flow cytometry.
Software	FACSDiva (BD Biosciences) was used for data acquiring FlowJo v.10.1 was used for data analysis.
Cell population abundance	In general, sorting led to > 95% purity as indicated by FACS. Purity was controlled by running the post-sorted sample by FACS.
Gating strategy	Lymphocytes gate was analyzed depending on distinguished FSC vs. SSC properties. Singlets were then selected (FSC-W vs FSC-H then SSC-W vs SSC-H). When applicable, dead lymphocytes were excluded (using Sytox® blue dead cell stain; Life Technologies, or Fix viability dye) and the living cells were further analyzed according to their surface or intracellular protein stains. At least 1x106 cells were used per staining and 100,000 total events were collected from each sample when possible. Isotype antibodies or unstained cells were used as negative controls to indicate the boundaries between negative and positive populations.<br>The sample preparations from different organs or acquired at different timepoints were treated in a comparable way and were acquired using the same settings.

- [x] Tick this box to confirm that a figure exemplifying the gating strategy is provided in the Supplementary Information.
